# Oxygen Evolution Reaction in Energy Conversion and Storage: Design Strategies Under and Beyond the Energy Scaling Relationship

**DOI:** 10.1007/s40820-022-00857-x

**Published:** 2022-04-28

**Authors:** Jiangtian Li

**Affiliations:** grid.420282.e 0000 0001 2151 958X U.S. Army Combat Capabilities Development Command Army Research Laboratory, 2800 Powder Mill Road, Adelphi, MD 20783 USA

**Keywords:** Oxygen evolution, Energy conversion and storage, Scaling relationship, Catalytic descriptors, Lattice oxygen oxidation

## Abstract

The oxygen evolution reaction (OER) is the essential module in energy conversion and storage devices such as electrolyzer, rechargeable metal–air batteries and regenerative fuel cells. The adsorption energy scaling relations between the reaction intermediates, however, impose a large intrinsic overpotential and sluggish reaction kinetics on OER catalysts. Developing advanced electrocatalysts with high activity and stability based on non-noble metal materials is still a grand challenge. Central to the rational design of novel and high-efficiency catalysts is the development and understanding of quantitative structure–activity relationships, which correlate the catalytic activities with structural and electronic descriptors. This paper comprehensively reviews the benchmark descriptors for OER electrolysis, aiming to give an in-depth understanding on the origins of the electrocatalytic activity of the OER and further contribute to building the theory of electrocatalysis. Meanwhile, the cutting-edge research frontiers for proposing new OER paradigms and crucial strategies to circumvent the scaling relationship are also summarized. Challenges, opportunities and perspectives are discussed, intending to shed some light on the rational design concepts and advance the development of more efficient catalysts for enhancing OER performance.

## Highlights

- Catalytic descriptors for oxygen evolution reaction under scaling relationship are comprehensively reviewed.
- New oxygen evolution paradigms and design strategies aiming to circumvent the adsorption energy scaling relationship are summarized.
- Challenges and perspectives for further improving oxygen evolution activity are discussed.

## Graphical abstract

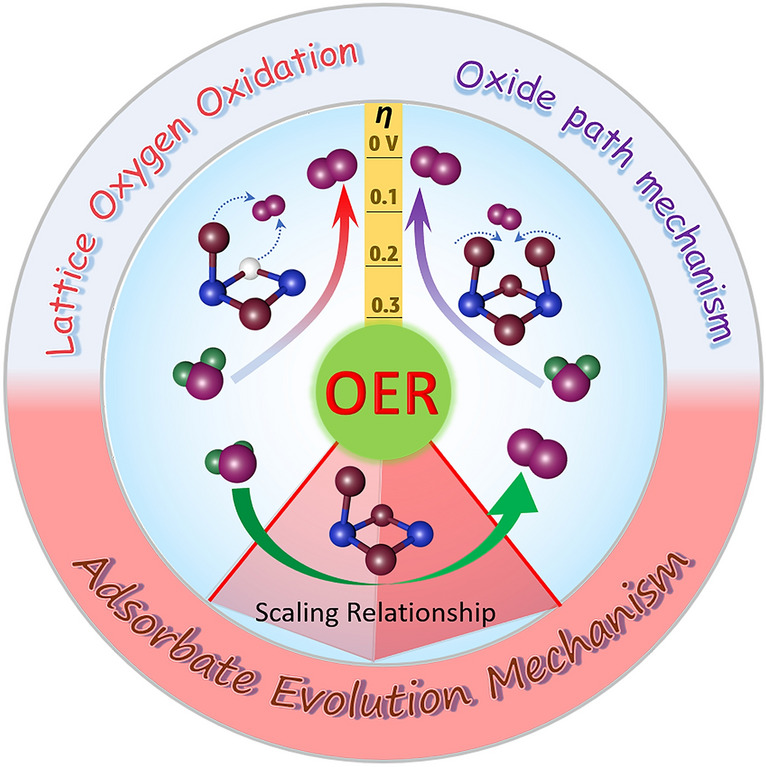

## Introduction

**Fig. 1:**
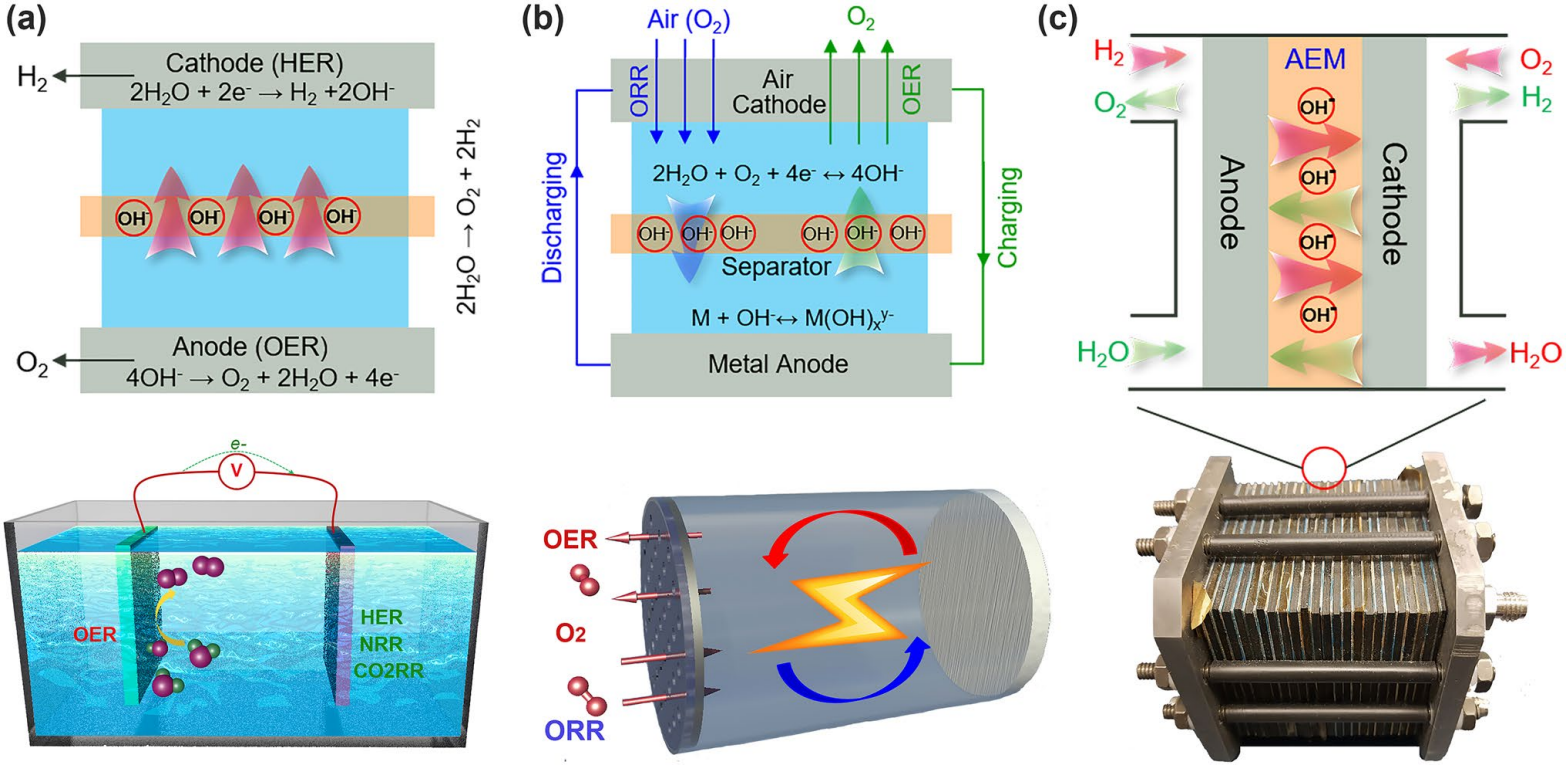
The OER in electrochemical energy conversion and storage devices. **a** Electrolyzers for small molecules’ electrolysis, **b** metal–air batteries, and **c** regenerative fuel cells

The ever-increasing energy demands and climate change along with the exhausting fossil fuels require the modern society urgently to seek renewable and sustainable energy sources, predominantly the solar and wind. However, the distributed and intermittent nature of such renewable energy sources presents a formidable challenge toward their effective utilization [1–3]. Exploring high-performance energy conversion and storage (ECS) devices, such as small molecule (water, carbon dioxide and nitrogen) electrolyzers, rechargeable metal–air batteries, and regenerative fuel cells, that can harvest, convert and store the renewable energy in chemicals and then reconvert at the point of need, is therefore of essential importance but remains a great scientific challenge [4–12]. The oxygen evolution reaction (OER) is the essential module in these ECS devices since it supplies electrons required for electrochemical conversion cycles between renewable electricity and chemical fuels [13–16]. In an electrolyzer for chemical fuel generation by small molecules’ electrolysis, the OER occurs at the anode (Fig. 1a) [2]. Whereas, the OER proceeds on the cathode in metal–air battery (MAB in Fig. 1b); its activity and stability immediately determine the charging and discharging performances of MAB devices [17–19]. A regenerative fuel cell which operates in two modes of hydrogen production (electrolyzer cell mode) and power production (fuel cell mode) is able to provide an economical means for efficient long-term energy storage and on-demand conversion back to electrical energy only with the participation of powerful oxygen electrolysis (Fig. 1c) [20, 21]. Improving the OER efficiency is therefore crucial to realize a close-looped clean energy infrastructure based on the conversion and storage of renewable energy [22].

The OER that involves the transfer of four electrons and the formation of multiple intermediates is challenging. Electrocatalysts play a key role by facilitating the required electron transfer, as well as the formation and rupture of chemical bonds [14, 23]. Nowadays, the state-of-the-art OER catalysts are the precious metal Ir/Ru-based materials. Besides the high cost and low reserve, the benchmark OER catalyst IrO_2_ has a regular overpotential *η*_10_ of around 300–400 mV (at the current density of 10 mA cm^−2^), which, however, is still far from an ideal OER catalyst in terms of activity [14]. The first-row transition metal oxide (TMO) electrocatalysts (such as perovskite, spinels, rock salt, and rutile) sparked intensive interests for catalyzing the OER with impressive activity, thanks to their low cost and superior oxidation resistance in alkaline electrolyte and, particularly upon high oxidation potentials during the OER [19, 24–28]. Nonetheless, no one TMO can fully satisfy the requirements for commercial operations. Again, the grand challenge is to develop advanced electrocatalysts with high activity and stability based on non-noble metal materials to enable the widespread penetration of clean ECS technologies [14, 19, 29].

Central to the rational design of advanced and high-efficiency catalysts is the understanding of quantitative structure–activity relationships, which correlate the catalytic activities with structural and electronic parameters [30]. In the context of OER, the large intrinsic overpotential is actually at the very root of why widespread deployment of ECS devices is still largely hampered. The origin of high overpotential for the OER is the strong scaling relations among the three oxygen-containing intermediates (*OH, *O, and *OOH) that impose a minimum intrinsic overpotential of about 0.37 V on all OER catalysts. Under this scaling relationship, intensive efforts have been made to propose catalytic descriptors such as *d*-band center, bulk O-2*p* band center, *e*_g_ occupancy, metal–oxygen covalency, electronegativity, coordination number, outer electron numbers, bulk thermochemistry and so on, aiming to bridge the structures and catalytic behaviors of catalysts and optimize the activity to reach the apex of the volcano plot. On the other hand, some recent advances have been achieved to circumvent the scaling relation limit for further improving the OER activity beyond the state-of-the-art. It is essential to have an in-depth understanding of the material-property relationships before designing advanced materials and structures with superior electrocatalytic properties [1, 31].

In view of this necessity, this paper comprehensively summarizes the benchmark descriptors for OER electrolysis to clarify the correlations between structures and catalytic behaviors of catalysts, intending to shed light on the origins of the electrocatalytic OER activity and further contribute to advancing rational catalyst design. This paper starts with the introduction of the conventional adsorbate evolution mechanism (AEM) and identify the intrinsic origin of large overpotential for the OER imposed by adsorption energy scaling relations. Then, catalytic descriptors under the scaling relationship are fully reviewed and standardized with the most recent research advances. Particular emphasis is placed on the newly proposed strategies to circumvent the scaling relationship toward designing novel catalysts with superior catalytic activities. The frontiers in this field such as lattice-oxygen participated OER and magnetic-dependent OER are highlighted. Based on the discussion above, a brief summary and outlook on the designing efficient OER catalysts are proposed and offered at the end.

## OER Under Scaling Relations

In this section, we introduce the fundamental principle of the OER under scaling relationship and provide an insight into what gives rise to the high intrinsic overpotential and what descriptors have been proposed to rationalize the correlation between catalytic activity and electronic structures. Since TMOs are regularly employed given their low cost and stability in alkaline media, the alkaline OER mechanism is adopted in this paper.

### Fundamental Principles of Adsorbate Evolution Mechanism

The well-acknowledged OER process is based on the adsorbate evolution mechanism (AEM), by which the oxygen-containing adsorbates undergo catalytic reactions on the surface-active TM cations of the catalyst. Therefore, the AEM could be regarded as the redox reactions of metal cations. On the whole, the oxygen generation in alkaline media follows Eq. 1, which is accompanied with the transfer of four electrons.

1 $$4{\text{OH}}^{ - } \to {\text{ O}}_{2} + \, 2{\text{H}}_{2} {\text{O}} + 4{\text{e}}^{ - }$$

**Fig. 2:**
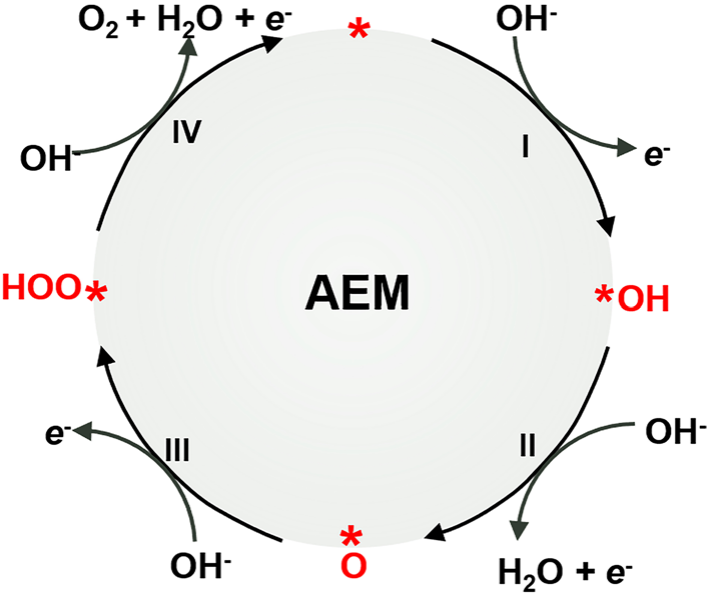
The OER catalytic cycle based on AEM. Here, * represents the surface-active TM cation. The four elementary steps I, II, III, IV correspond to Eqs. 2–5

Theoretically, this process has a standard electrode potential (*E*^0^) of 0.401 V versus the standard hydrogen electrode (SHE) and a formal potential ($E^{0\prime }$) of 1.229 V versus the reversible hydrogen electrode (RHE) [22, 23]. In practical operations, however, it proceeds through multiple elementary steps and, thus, requires considerably high applied overpotential ($\eta_{{{\text{OER}}}} = E - E_{{{\text{O}}^{2} {\text{/OH}}^{ - } }} > 0.3 \;{\text{V}}$) [22, 23, 32–34]. Of note, the high applied potential to drive the OER usually leads to the oxidation of catalysts, thereby metal oxides are considered as the most potent catalysts for the OER regarding the long-term stability [35]. In the light of the computational prediction, the oxygen generation cycle involves four concerted electron-transfer steps as shown in Fig. 2 [20, 32]. The OER starts with adsorption of OH^−^ at an active site (*) to generate *OH radical (Step I, Eq. 2). Then, *OH deprotonates to produce *O, which is accompanied with the release of an electron and a water molecule (Step II, Eq. 3). Thereafter, the nucleophilic attack of OH^−^ on *O yields the intermediate *OOH (Step III, Eq. 4). Finally, a further proton-coupled-electron transfer process results in the generation of one oxygen molecule as well as a free active site (Step IV, Eq. 5) [36].

2 $$^{*} + {\text{OH}}^{ - } \to ^{*}{\text{OH}} + {\text{e}}^{ - }$$

3 $$^{*}{\text{OH}} + {\text{OH}}^{ - } \to ^{*}{\text{O}} + {\text{H}}_{2} {\text{O}} + {\text{e}}^{ - }$$

4 $$^{*}{\text{O}} + {\text{OH}}^{ - } \to ^{*}{\text{OOH}} + {\text{e}}^{ - }$$

5 $$^{*}{\text{OOH}} + {\text{OH}}^{ - } \to ^{*} + {\text{O}}_{2} + {\text{H}}_{2} {\text{O}} + {\text{e}}^{ - }$$

Any step in the process could restrain the overall OER performance. The adsorption, dissociation and desorption of oxygen-containing intermediates play a decisive role herein [35]. Though the multiple adsorbate-based steps have not been fully visualized experimentally, the common consensus is that the interactions between the catalyst surface and adsorbates unambiguously govern the OER catalytic activity. Accordingly, a systematic understanding of the interaction between the active sites and the oxygen-containing intermediates during the reaction is critical for developing more efficient OER electrocatalysts [34].

### Scaling Relations

**Fig. 3:**
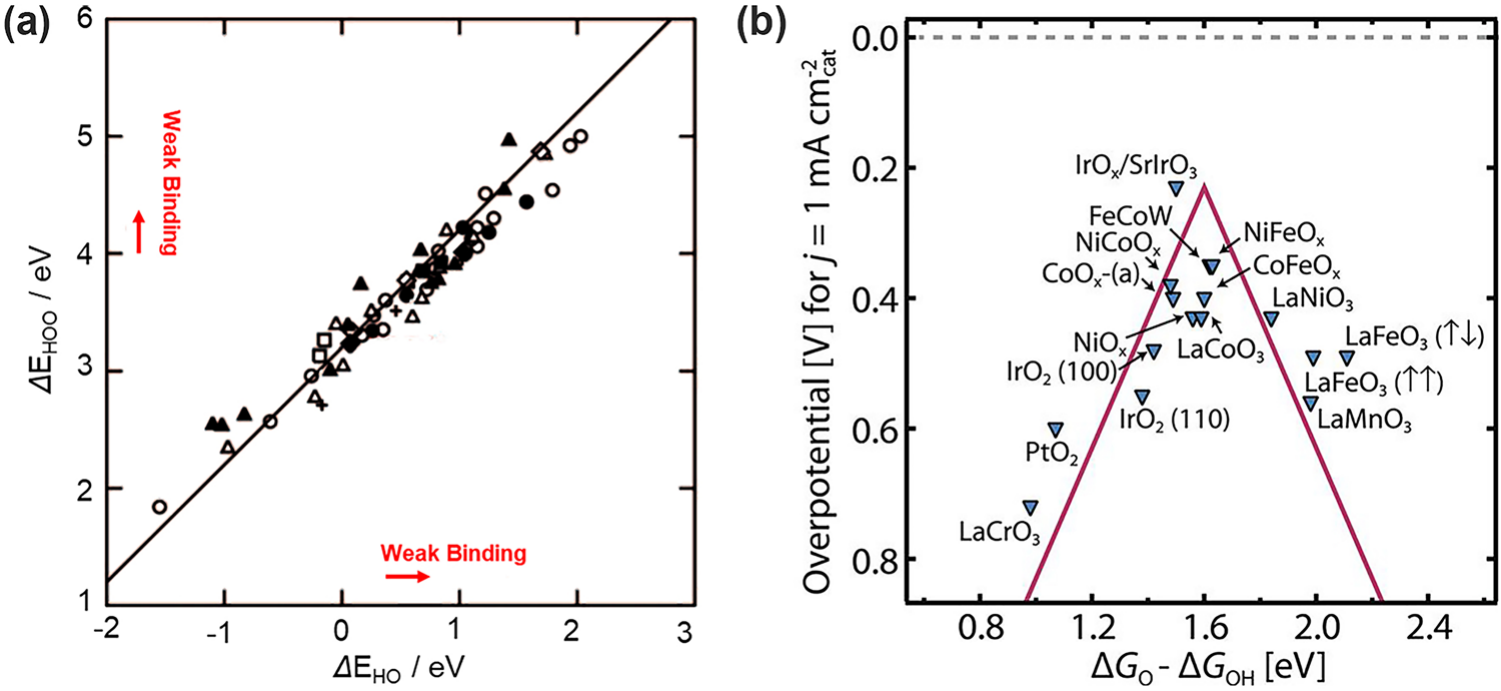
The adsorption energy scaling relations between oxygen-containing intermediates. **a** Plot of adsorption energies of HOO* and HO* on metal oxides. Hollow symbols represent the adsorption energy on the clean surfaces: perovskites (circle), rutiles (triangle), Mn_*x*_O_*y*_ (square), anatase (diamond), Co_3_O_4_ (+). The solid symbols represent the adsorption energies on high coverage surfaces [37]. Copyright © 2011 Wiley–VCH. **b** The volcano relationship between Δ*G*_O*_–Δ*G*_HO*_ and OER activity for major oxides [14]. Copyright © 2017 American Association for the Advancement of Science

The electrocatalytic activity is to a large extent determined by the binding strength between the reaction intermediates and the catalyst surface, that is, the binding energy. Empirically, an ideal catalyst should be able to adsorb the intermediate with an optimal binding energy that is neither too strong nor too weak as stated by the Sabatier principle [1, 37]. Nowadays, advances in density functional theory (DFT) calculations make it possible to quantitatively determine the binding energy [37]. As schemed above in Fig. 2, the conventional AEM consists of four consecutive electron-transfer steps and three oxygen-containing intermediates, *OH, *O and *OOH, which are all adsorbed on the surface through O atoms. Under this circumstance, the binding energies of *OOH (*E*_HOO*_), *O (*E*_O*_), and *OH (*E*_HO*_) are linearly correlated [37–39]. *E*_HOO*_/*E*_HO*_ shows a slope of approximately 1 and an intercept of 3.2 eV (Fig. 3a) [37]; analogously, both *E*_HOO*_/*E*_O*_ and *E*_HO*_/*E*_O*_ have slopes close to 0.5, since O* is double bonded to the surface, and HO* and HOO* are bound with a single bond [38–40]. This correlated binding energy relationship universally occurs on metal and metal oxide surfaces (including perovskite, spinel, rutile, rock salt, and bixbyite oxides and so on) independent of the binding strength to the surface, and regardless of binding sites. On the surfaces that bind oxygen too strongly, the potential is limited by the formation of *OOH species; whereas for the surfaces that bind oxygen too weakly, the potential is limited by the oxidation of *OH [37]. This gives rise to a volcano-shaped relationship between catalytic activity and the oxygen adsorption energy (Fig. 3b) [14, 16]. The catalysts that locate on the top of the volcano always have balanced binding energies and thus, demonstrate better OER performance. This paradigm has been acknowledged as a universal descriptor to successfully interpret and predict the OER activity of various catalysts. One can optimize the catalytic activity by approaching Δ*G*_O*_- Δ*G*_HO*_ toward the apex of the volcano plot [41].

However, the problem raised by the scaling relationship is leaving all adsorption energies interdependent with each other. In other words, the adsorption energy of an intermediate cannot be freely tuned without affecting another [36, 37, 39, 41, 42]. Most importantly, the established scaling relationship (Δ*G*_OOH_ = Δ*G*_OH_ + 3.2 V) brings about an intrinsic overpotential as large as (3.2–2.46) eV/2e^−^ = 0.37 V [2, 37]. This means that even the best OER catalysts still have a minimum theoretical overpotential of about 0.3–0.4 V, which sets up a limit for OER performance that cannot be further bypassed theoretically [41]. Novel paradigm to circumvent the scaling relationship is thus in urgent demand for OER electrocatalyst design.

### Descriptors Under Scaling Relationship

A catalytic descriptor is established to identify the relations between the catalytic behavior and the catalyst, which should not only be able to explain and screen the activity trend but also allow for the exploration of more active catalysts. In the past decade, intensive efforts have been made to discover catalytic descriptors for electrocatalytic OER in terms of bulk and surface electronic structures [1, 30, 43].

#### d-Band Theory

**Fig. 4:**
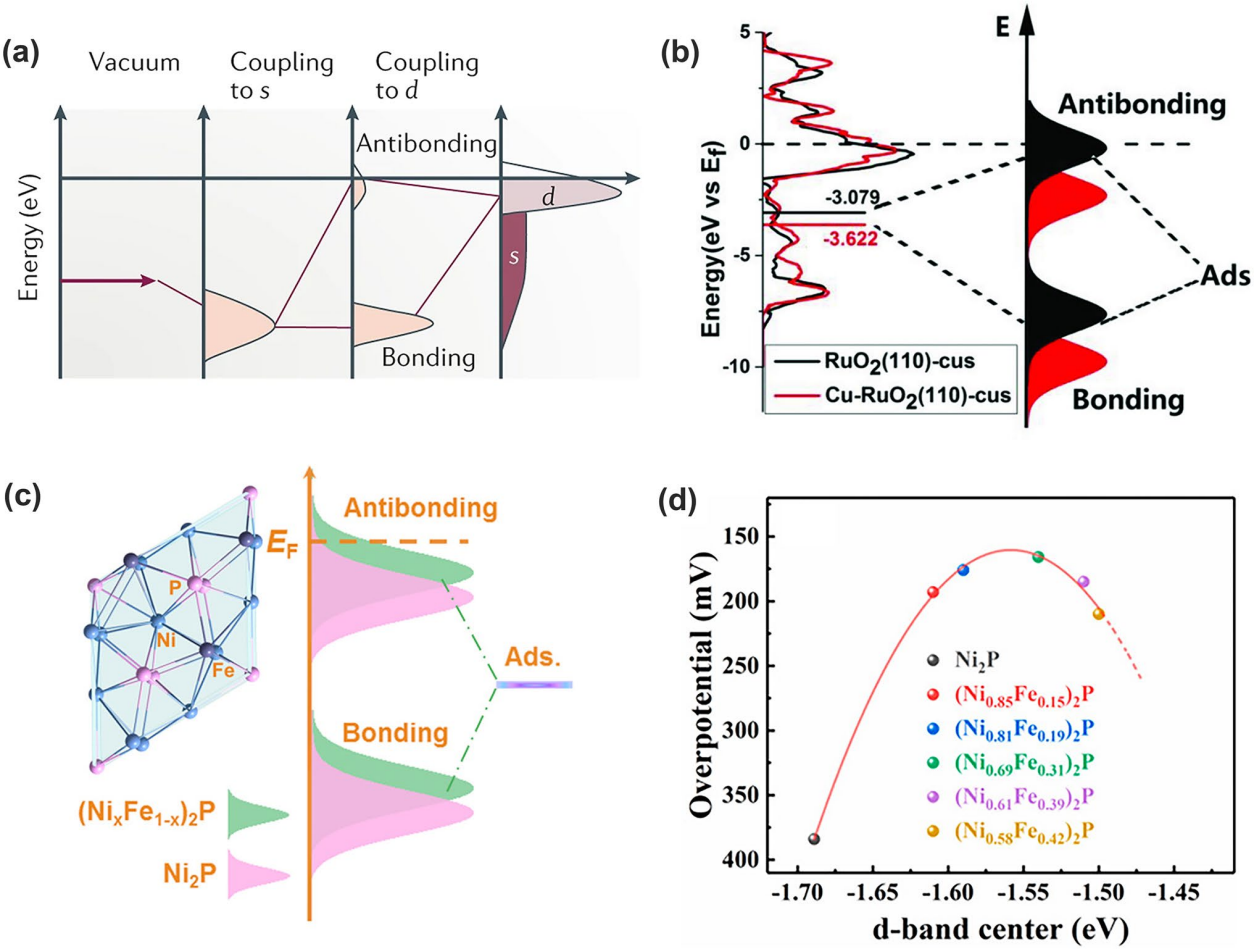
**a** Schematic illustration of bond formation at a transition-metal surface. The lower the *d* states are in energy relative to the E_F_, the more filled the antibonding states and the weaker the adsorption bond [45]. Copyright © 2019 Springer Nature. **b** The density of states (DOS) plots of RuO_2_, Cu-RuO_2_, and the corresponding schematic illustration of bond formation between the reaction surface and adsorbate [49]. Copyright © 2018 Wiley–VCH. **c** *d*-band centers of Ni_2_P and Fe-doped Ni_2_P; **d** Relationships of the calculated *d*-band centers and experimental overpotential of Fe-doped Ni_2_P [50]. Copyright © 2020 American Chemical Society

The relationship between the electronic structure and the catalytic activity of TMs was first established with *d*-band theory in 1990s by Hammer and Nørskov [44]. The *d*-band theory describes the bond formation at a TM surface, as illustrated in Fig. 4a. To improve the description of the energy level of *d* band, the *d*-band center (i.e., the average energy of the band) was generally adopted. Upon adsorption on the TM surface, the valence states of the adsorbate couple with the TM *s* states and become broadened and shift downward, which then continue to interplay with the TM *d* states and form the filled bonding states and partially occupied antibonding states [45]. The antibonding states are above the *d* states, and its filling governs the bond strength in terms of the distance from the band center to the Fermi level (*E*_F_) [44]. In general, the higher the *d* states are in energy relative to the *E*_F_, the higher in energy the antibonding states are and the stronger the bond [31, 46, 47].

The *d*-band model has been widely adopted to understand bond formation and trends in reactivity among the TMs and their alloys. The facet, alloying, defects, strain and so forth greatly impact the *d* states and thus alter the reactivity [44, 45]. In context of the OER, the free-energy descriptor requires balanced adsorption and desorption of oxygen-containing intermediates, which thus necessitates an optimal *E*_d_ energy level for electrocatalyst to maximize the OER activity. Analogous to the alloying effect for TMs, doping TM-based compounds is the most effective pathway to impact the *d* states [46, 48]. The overly strong Ru–O bond in RuO_2_ regularly gives the high free energy of the rate determining step (RDS). With Cu being doped into RuO_2_ lattice, the *d*-band center shifts far away from the* E*_F_, leading to lowered antibonding states and weakened Ru–O bond strength (Fig. 4b) [49]. Conversely, the largely low *E*_d_ energy level of Ni_2_P (that is, the weak adsorption energy) makes oxygen-containing intermediates difficult to adsorb on the surface. Chen and coworkers found that Fe doping could raise the *E*_d_ energy levels closer to the E_F_ (Fig. 4c) and thus, lifted the antibonding energy states and strengthened the interaction between adsorbates and catalyst surface, which enhanced the adsorption ability for intermediates during the OER process [50]. It is noted that a volcano-shaped OER performance against the *d*-band center was obtained by varying Fe concentrations (Fig. 4d). This means that a highly active OER catalyst should have an optimal *E*_d_ energy level to balance the adsorption and desorption of intermediates (*OH, *O, and *OOH) [50].

#### Bulk O-2p Band Center

**Fig. 5:**
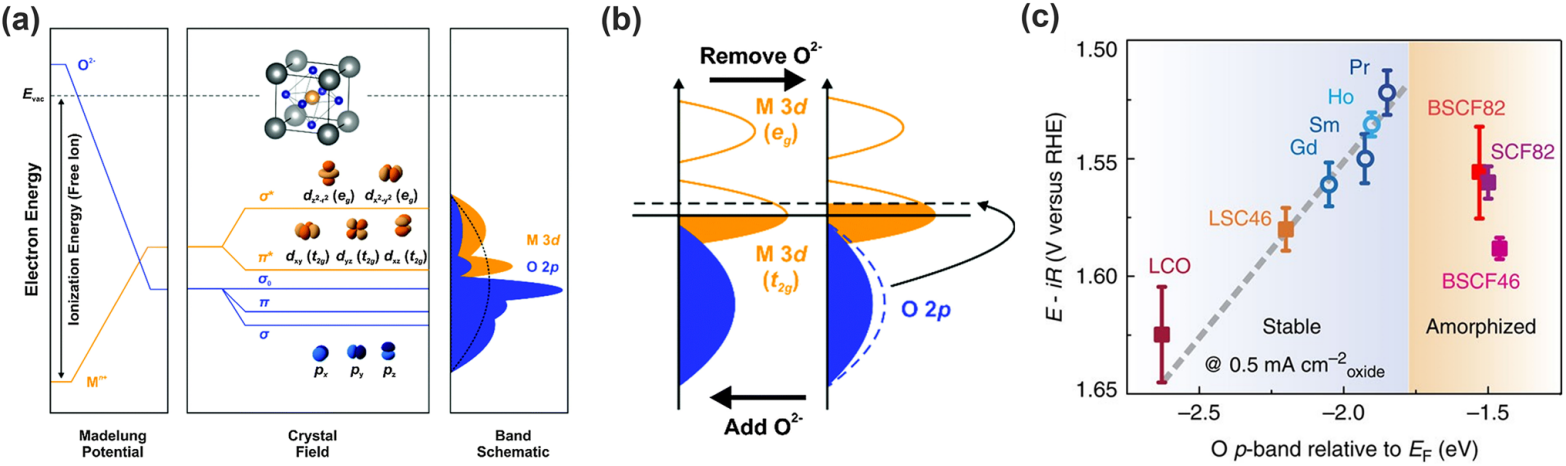
Bulk O 2*p*-band center relative to Fermi level (*ε*_O-2p_) as a descriptor for OER. **a** Physical origin of shifts in constituent ion orbitals for oxides with octahedral oxygen coordination around transition metal ions. The dashed line represents the energy of free vacuum. **b** Schematic illustration of the density of states of perovskite oxides, showing the transition metal 3*d* and O 2*p* bands [51]. Copyright © 2015 Royal Chemical Society. **c** Correlation between the iR-corrected potential at 0.5 mA cm^−2^ and the *ε*_O-2p_ (eV) of (Ln_0.5_Ba_0.5_)CoO_3−*δ*_ with Ln = Pr, Sm, Gd and Ho [4]. Copyright © 2013 Springer Nature

Compared to TMs, electronic structure of TM cations in oxides undergoes significant changes upon being surrounded by an array of point charges generated by oxygen ions, where the five originally degenerated *d* orbitals lose the energy degeneracy (Fig. 5a). In an octahedrally coordinated field, the *d* orbitals split into two *e*_g_ orbitals at the higher energy level and three *t*_2g_ orbitals at lower energy level. The *e*_g_ doublets have strong overlap with O 2*p* orbitals and generate* σ*-*bonding* and  σ*-*antibonding* states. Whereas, the *t*_2g_ triplets demonstrate weak overlap with O 2*p* orbital and form π-bonds and π*-antibonds. The molecular orbitals become bands in oxide crystals due to the translational symmetry of the unit cell, denoted as M *d* band and O 2*p* band [51]. The bonds develop through the hybridization of M *d* orbitals and O 2*p* orbitals, which could be flexibly adjusted given the abundant choices of TM ions and their oxidation states. Moreover, the electronic structure of oxides can also be influenced by the spin states, that is, the relative occupancy of *e*_g_ and *t*_2g_ states, which has been shown to influence electronic conductivity, thermal expansion, bulk modulus and catalytic activity [51]. The variable tunability on TM ions like number of *d* electron, oxidation state, electronegativity, coordination numbers, and arrangement and nature of ligands has sparked intensive explorations of useful descriptors on screening and predicting the efficient OER catalysts.

Morgan and coworkers demonstrated that the bulk O 2*p*-band center relative to the Fermi level (ε_O-2p_) correlated strongly with the oxygen surface kinetics and vacancy formation energy in perovskites [52], which later indicated its effectiveness in explaining the associated energetics and kinetics of all oxygen point defects in Ruddlesden–Popper oxide and polycrystalline perovskite materials [53, 54]. This descriptor can be understood in a rigid band model (Fig. 5b). Typically, removing oxygen from the lattice corresponds to moving electrons from the O 2*p* states to the Fermi level, which leads to the uplift of the Fermi energy level. Conversely, moving electrons from Fermi level to O 2*p* band is equivalent to the addition of oxygen [4, 52]. The O 2*p*-band center reflects differences in the Fermi energy of the oxide, as the absolute energy of the O 2*p*-band largely depends on the Madelung potential and oxygen electron affinity [43, 51], which meanwhile can be regulated by varying the electronegativity and oxidation states of the transition metals [51, 55, 56]. Different to the *d* band model, the O 2*p* band demonstrates more electron delocalization and thus is able to more accurately capture the electronic structure characteristics in oxides [57, 58].

Grimaud and coworkers correlated the high activity and stability of some double perovskites ((Ln_0.5_Ba_0.5_)CoO_3-δ_, Ln = Pr, Sm, Gd, and Ho) with O 2*p*-band center being neither too close nor too far from the Fermi level (Fig. 5c) [4]. Moving the O 2*p*-band center closer to the Fermi level increased OER activities, while the overlifting (that is, too close to the Fermi level) led to the decreased stability because of the surface amorphization (BSCF, BSCF46 and SCF) [4]. Similarly, the amorphous surface after OER was also observed in BSCF82 with a high O 2*p* band center [59]. It was believed that the high O 2*p* band centers increase the oxygen-vacancy concentration due to much lower oxygen vacancy formation energy, which accelerates the surface oxygen exchange kinetic and facilitates lattice oxygen migration, thus leading to surface amorphization during the OER process [59, 60].

#### ***e***_g_ Occupancy

**Fig. 6:**
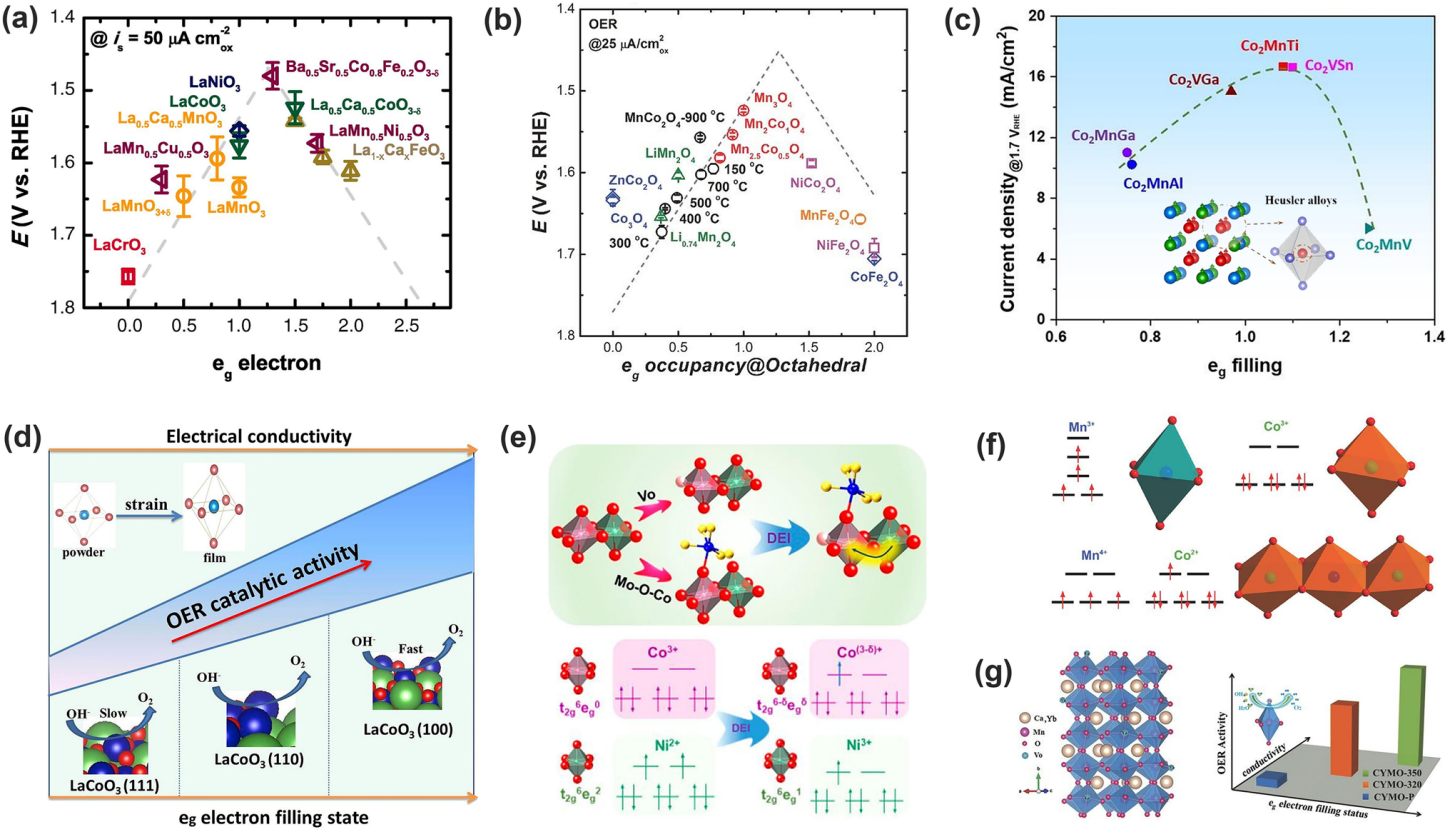
Correlation between *e*_g_ occupancy and OER activity of TMOs, and strategies to regulate the *e*_g_ occupancy. OER activity as a function of eg occupancy of the active center at octahedral site on **a** perovskite oxides [63], **b** spinel oxides [65], and **c** Heusler alloys [70], respectively. Copyright © 2011 American Association for the Advancement of Science. Copyright © 2017, 2021 Wiley–VCH. **d** The relationship between OER activity and spin configuration of differently oriented LCO films [73]. Copyright © 2017 Elsevier. **e** Double exchange interaction between Co and Ni at octahedral sites in spinels and the transitions of the *e*_g_ occupancy [25]. Copyright © 2020 American Chemical Society. **f** Charge redistribution between Mn and Co in Co-Mn containing spinels via superexchange interaction [75]. Copyright © 2018 Wiley–VCH. **g** Oxygen vacancy and doping induced the increased conductivity and *e*_g_ electrons filling status in perovskite CaMnO_3_ [77]. Copyright © 2015 Wiley–VCH

As stated above in the crystal field, the high-energy *e*_g_ orbitals readily reveal strong overlap with O 2*p* orbital in octahedral coordination, thus leading to maximized *p*–*d* hybridization with oxygen-containing intermediates. In 1970s, Matsumoto and co-workers found that the catalytic activity of perovskite oxides for oxygen reduction reaction was influenced by the overlap between the *e*_g_ orbital of the TM and the *p* orbital of oxygen adsorbates, and that the larger the overlap was, the higher the electrocatalytic activity [61, 62]. Suntivich and coworkers later theoretically and experimentally investigated the OER activities of more than 10 perovskite oxides against the *e*_g_ occupancy and gave a volcano relationship between them, that is, the *e*_g_ occupancy model (Fig. 6a) [63, 64]. The *e*_g_ occupancy descriptor was proposed upon the fact that the * σ*-interaction of *e*_g_ states dominates over the weaker π-interaction of the *t*_2g_ states [51, 63]; meanwhile, the number of electrons in  σ* states governs the binding strength of oxygen-containing intermediates with surface TM cations. The *e*_g_ occupancy of near unity (slightly bigger than 1) leads to optimum OER activity. If *e*_g_ < 1, the too strong M–O binding hinders the formation of M-OO^2−^; while if *e*_g_ > 1, the weak interaction lowers the formation rate of O–O bond in M-OOH^−^ [65]. The *e*_g_ occupancy model that only takes account of electrons in *e*_g_ orbital is different to those descriptors using all *d* electrons. [66, 67] With this model, the perovskite Ba_0.5_Sr_0.5_Co_0.8_Fe_0.2_O_3−*δ*_ (BSCF) was identified with the record intrinsic alkaline OER activity, though surface amorphization was later ascertained under OER conditions [4]. In case that oxides have similar *e*_g_ occupancy, the OER activity has to be judged with a secondary descriptor, like the electronegativity of the TM cation [64, 68].

Besides perovskites (ABO_3_) [69], the *e*_g_ occupancy model has been successfully applied to spinel oxides (AB_2_O_4_), mullites (AB_2_O_5_), and even Co_2_YZ-type Heusler compounds [70]. Xu and coworkers studied a series of MnCo_2_O_4_ spinels toward OER and oxygen reduction reaction (ORR) electrolysis, and identified the octahedrally coordinated Mn as the active sites. The correlation between OER/ORR activity (applied potential @ 25 μA cm^−2^_ox_) with *e*_g_ filling of Mn at octahedral site gave a volcano shape (Fig. 6b), with the summit located at the Mn valency of ≈ + 3 (that is, *t*_2g_^3^*e*_g_^1^), which consolidated the decisive role of electron orbital filling in metal oxide catalysts for oxygen electrolysis [65]. Of significance, Tüysüz and coworkers recently demonstrated that the *e*_g_ orbital filling of active Co sites could be precisely regulated by varying the Y and Z sites of the Co_2_YZ compounds, where the higher catalytic current was achieved for *e*_g_ orbital filling approaching unity [70]. This further supports the effectiveness of the *e*_g_ orbital filling model to modulate the M–O bonding strength towards more active OER catalysts (Fig. 6c).

Two parameters apparently regulate the *e*_g_ occupancy: the number of *d*-electrons (namely, the oxidation state) and the spin state. For example, Mn^3+^ (3*d*^4^) typically affords the *t*_2g_^3^*e*_g_^1^ configuration, while Mn^4+^ (3*d*^3^) adopts *t*_2g_^3^*e*_g_^0^ configuration. Fe^4+^ (3d^4^, *t*_2g_^3^*e*_g_^1^) and Ni^3+^ (3d^7^, *t*_2g_^6^*e*_g_^1^) prefer to afford *e*_g_^1^ configuration, while *e*_g_ orbitals in Fe^3+^ (3d^5^, *t*_2g_^3^*e*_g_^2^) and Ni^2+^ (3d^8^, *t*_2g_^6^*e*_g_^2^) are over-occupied. Meanwhile, the spin state is determined by the difference between the Hund exchange energy Δ_EX_ and crystal field splitting energy Δ_CF_. If Δ_EX_ > Δ_CF_, *d* electrons prefer to occupy the high-energy *e*_g_ orbitals rather than pair at *t*_2g_ orbitals; whereas, if Δ_EX_ < Δ_CF_, *d* electrons will first pair at the low-energy *t*_2g_ orbitals. One typical example is Co^3+^ (3*d*^6^) that has three different spin states: low-spin (LS, *t*_2g_^6^*e*_g_^0^), intermediate spin (IS, *t*_2g_^5^*e*_g_^1^) and high spin (HS, *t*_2g_^4^*e*_g_^2^). Under *e*_g_ occupancy model, regulating Co^3+^ at octahedral site to possess an intermediate-spin electron configuration is therefore expected to improve the OER activity. Substitution/doping with cations having different valences and electronegativities, vacancies, strain and even nanostructuring can effectivity tailor the oxidation state and spin state of octahedral metal centers [71, 72]. Wu and co-workers demonstrated a spin-state regulation method to optimize the OER activity by controlling the lattice orientation of LaCoO_3_ film. The different lattice-oriented LaCoO_3_ films brought different degrees of distortion of the CoO_6_ octahedron (namely, the strain), which induced a spin-state transition of cobalt from a low spin state (LS, *t*_2g_^6^*e*_g_^0^) to an intermediate spin state (IS, *t*_2g_^5^*e*_g_^1^) and thus better OER performance (Fig. 6d) [73]. Oxygen vacancy also plays a significant role in altering the *e*_g_ occupancy/spin states. In CoFe spinel oxide, electrons from the oxygen holes transferred to the neighboring cobalt sites, enabling the conversion of Co^3+^ from low spin to a stabilized higher spin state Co^(3−*δ*)+^ (*t*_2g_^6^*e*_g_^*δ*^, *δ* is close to 1.2) [74]. Our recent study demonstrated a novel strategy to modulate the spin states in NiCo_2_O_4_ spinel via double-exchange interaction between octahedrally coordinated Ni and Co (Fig. 6e) [25]. Double exchange interaction was triggered by the co-actions of constructing a covalent nanoheterojunction and creating oxygen vacancies (V_O_) in NiCo_2_O_4_ and proceeded with the equation Co^3+^ + *δ*Ni^2+^ → Co^(3−*δ*)+^ + *δ*Ni^3+^ (0 < *δ* < 1). As a result, the high-spin Ni^2+^ (*t*_2g_^6^*e*_g_^2^) was oxidized to low-spin Ni^3+^ (*t*_2g_^6^*e*_g_^1^), whereas the low-spin Co^3+^ (*t*_2g_^6^*e*_g_^0^) switched to intermediate spin Co^(3−*δ*)+^ (*t*_2g_^6−*δ*^ *e*_g_^*δ*^, *δ* close to 1). Both octahedral sites thus had optimal *e*_g_ occupancy close to 1 to readily improve OER activity. Doping with alien cations is another effective strategy to tune the electronic structure. Zhou and co-workers studied the Co–Mn containing spinel oxides ZnCo_*x*_Mn_2−*x*_O_4_ (*x* = 0.0–2.0) and found that the *e*_g_ occupancy of active Mn cations could be modulated through varying the Mn/Co ratio as a consequence of the superexchange effect between edge sharing CoO_6_ and MnO_6_ (Fig. 6f). The Mn/Co ratio of 0.43 gave the optimum *e*_g_ occupancy and the best catalytic activity [75]. In practical operations, multiple actions always work cooperatively to modulate the *e*_g_ occupancy. Guo and co-workers found that the introduction of oxygen vacancies in Yb-doped CaMnO_3_ through direct hydrogen treatment led to an increase in the *e*_g_ filling of Mn and the improvement of conductivity (Fig. 6g). The optimized Yb_0.1_Ca_0.9_MnO_3_ catalyst demonstrated exceptional OER activity, 100 times higher than that of the pristine CaMnO_3_, which originated from the synergistic effects of doping and oxygen vacancies [76, 77].

#### Metal–Oxygen Covalency

**Fig. 7:**
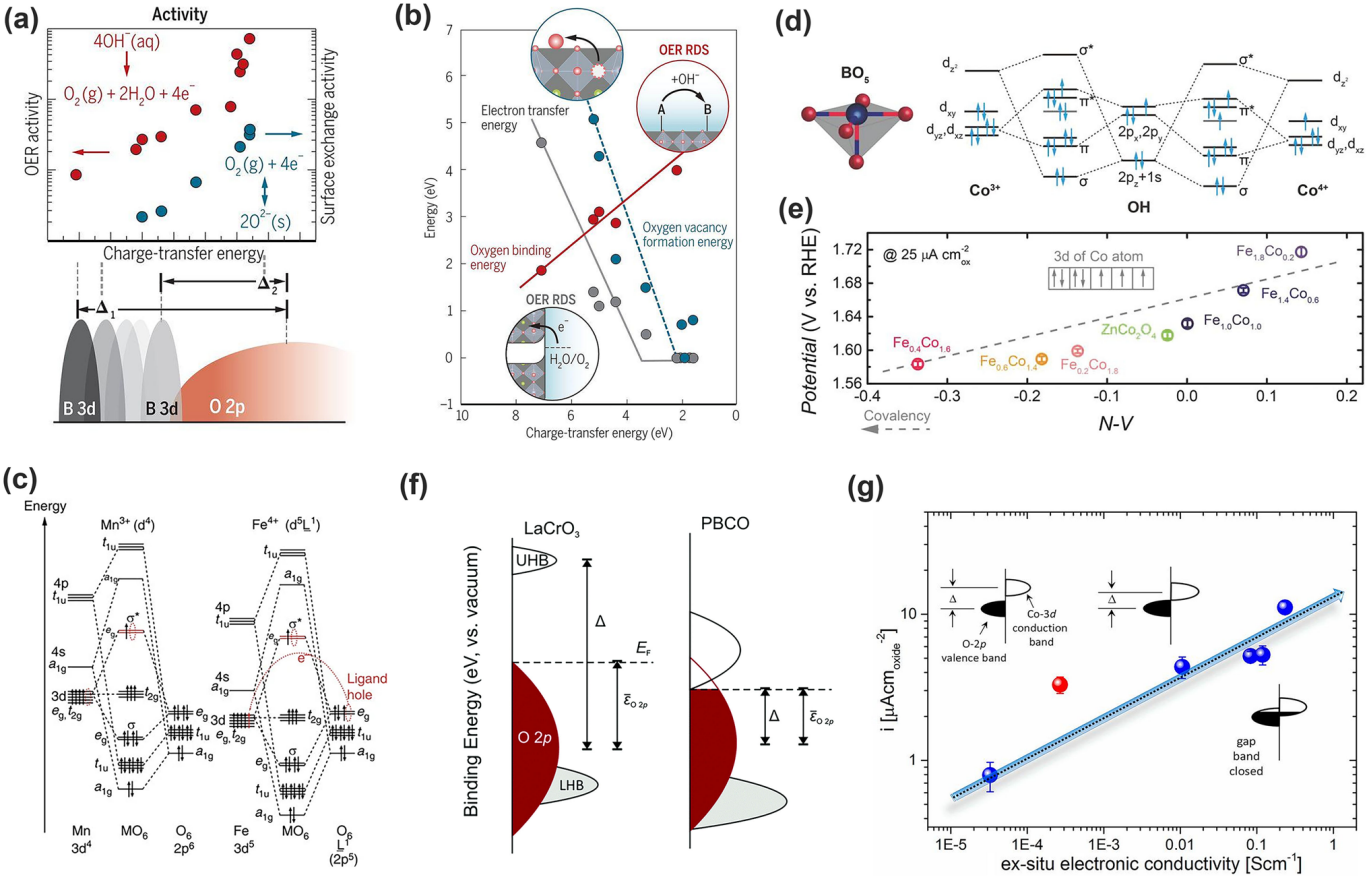
Correlation between Metal–oxygen covalency and OER activities. **a** Correlations between charge-transfer energy and OER activity (red), surface exchange activity (blue), and the OER active site identity in the 3d transition metals. **b** Relationship of charge-transfer energy to relevant energetics and rate-determining steps: oxygen vacancy formation (blue), oxygen binding energy (red), and electron transfer energy (gray) [78]. Copyright © 2017 American Association for the Advancement of Science. **c** Schematic illustration of molecular orbitals for regular Mn^3+^O_6_ and Fe^4+^_O6_ octahedra. The Mn^3+^ and Fe^4+^ ion 3*d*-orbital energy levels are higher and lower than those of the O 2*p* orbitals, respectively [83]. Copyright © 2015 Springer Nature. **d** Molecular orbital diagrams of Co–OH (Co^3+^ and Co^4+^) bonding at the surface of spinel oxides. **e** OER activity as a function of the N–V parameter [84]. Copyright © 2018 Wiley–VCH. **f** Scheme for the charge-transfer energy: the relative energies of TM 3d and O 2p valence electronic states [79]. Copyright © 2017 Royal Society of Chemistry. **g** Current density (μA cm_oxide_^−2^) as a function of the ex situ electronic conductivity of the La_1−*x*_Sr_*x*_CoO_3_ series; the red circle represents the SrCoO_2.5_ [88]. Copyright © 2015 American Chemical Society. (Color figure online)

Despite the great success of *e*_g_ occupancy model as a useful activity descriptor, few weaknesses impede its universal effectiveness. First, it is technically difficult to ascertain the *e*_g_ filling of active metal sites responsible for catalysis where the surface spin state is not well identified. The representative example is cobalt with three possible spin sates that have raised extensive debate [78]. Second, the *e*_g_ occupancy model fails to screen the catalysts having the similar *e*_g_ occupancy but different catalytic activities like LaBO_3_ (B = Mn, Co, Ni) [30]. Third, surface amorphization observed in some OER catalysts like BSCF is far beyond the explanation of *e*_g_ occupancy. The major reason is ascribed to that the *e*_g_ occupancy descriptor was established on the ionic model and was unable to effectively capture the metal–oxygen covalency or the sharing of electrons along the metal–oxygen bond [78]. Shao-Horn and coworkers therefore introduced the metal–oxygen covalency (charge transfer energy) as a more powerful descriptor because the metal–oxygen bonds in oxides have mixed ionic-covalent character, that is, the energetic similarity (covalency) and spatial overlap (hybridization) of metal 3*d* orbitals and O 2*p* states [78]. The metal–oxygen covalency has been shown to influence catalytic activities. It correlated linearly with experimental OER activity, surface exchange activity (such as the oxygen vacancy formation energy, oxygen binding energy, and electron transfer barrier associated with OER), as well as the stability in alkaline solution (Fig. 7a) [71, 78, 79]. This model well explained the activity of LaBO_3_ (B = Mn, Co, Ni) with an order of Ni > Co > Mn in terms of the TM electronegativity that is linearly correlated with M–O covalency [30, 64, 68]. Regarding the stability, there exists a positive correlation between covalency and oxygen vacancy formation: too high of a driving force for oxygen vacancy formation leads to structural loss of the perovskite phase (Fig. 7b) [78]. As a result, an optimum covalency, as measured by the O 2*p* band center model, gave rise to both high activity and stability in Pr_0.5_Ba_0.5_CoO_3-*δ*_ (Fig. 5b). The covalency and the hybridization between M 3*d* and O 2*p* can be tuned by varying the electronegativity of the metal ions through the choice of TM and the oxidation state. In practice, metal substituents with higher electronegativity than the parent metal could reduce the energy of the *d* band, leading to enlarged metal–oxygen covalency [79, 80]. The incorporation of other low-valent metals or metal vacancy also increases the oxidation state of the metal, thereby lowering the metal 3*d* states into O 2*p* states and enlarging the metal–oxygen covalency [81, 82]. With this concept, Yagi and coworkers reported that the Fe^4+^-based quadruple perovskite CaCu_3_Fe_4_O_12_ with high OER activity [83]. Compared with Mn^3+^, the 3*d*-orbital energy levels of Fe^4+^ ion are lower than that of the O 2*p* orbitals, leading to enhanced metal–oxygen covalency (Fig. 7c). On the other hand, the large overlap between Cu (Fe) *e*_g_ and O 2*p* orbitals in the square-planar (octahedral) coordination improves the structural stability of CaCu_3_Fe_4_O_12_. The covalent bonding network incorporating multiple Cu^2+^ and Fe^4+^ transition metal ions significantly enable the long-life structural stability and exceptional OER activity in CaCu_3_Fe_4_O_12_ [83].

The metal–oxygen covalency readily proved its effectiveness in other metal oxides like spinels [84], rocksalt oxide [85], and Ruddlesden–Popper oxide [86]. Xu and coworkers did intensive work on spinel oxides for OER [84, 87]. They presented a systematic study of spinel ZnFe_x_Co_2-x_O_4_ oxides toward the OER, and found that Fe substitution (10–30 at% of Fe) promoted the formation of Zn vacancies and Co^4+^. Co^4+^ has a low energy 3*d* state and would produce higher hybridization due to the shortened energy distance between Co 3*d* and O 2*p* states, namely an enlarged Co–O covalency, which dominate the distinct OER activity (Fig. 7d–e) [84]. Later, with metal–oxygen covalency they further explained that the octahedral geometry in spinel oxides was more catalytically critical than the tetrahedral one [87].

Different to the O 2*p*-band center model that scales with the metal–oxygen covalency for semimetal oxides, but not for semiconducting oxides, the metal–oxygen covalency model tracks the energy difference between the M 3*d* and O 2*p* band centers (*ε*_3d-2p_), i.e., the charge transfer energy (Δ) [79]. The charge transfer energy plays a pronounced role in tuning the electronic properties of oxides that can impact the OER kinetics and mechanism. This has been widely confirmed in experiments with different TM substitution by varying the electronegativity and oxide states. Hong and coworkers found that reducing the charge transfer energy could greatly enhance the OER activity [79]. As shown in Fig. 7f, the reduced charge transfer energy Δ in PBCO relative to LaCrO_3_ leads to increased electrical conductivity and improved OER activity [79]. Sr doping into LaCrO_3_ (La_1−x_Sr_x_CoO_3_) raised the realignment of Co–O–Co bonds and the oxidation of the Co cations, which enlarged the overlap between the occupied O 2*p* valence bands and the unoccupied Co 3*d* conduction bands, and improved the conductivity (Fig. 7g) [88]. It is significantly noted that the changes in metal–oxygen covalency are always accompanied with the variations in the O 2*p* band, *d* band and even the spin states of the involved TMs owing to their close relationships. Duan and coworkers found that Fe substitution in LaCoO_3_ resulted in the transition of Co^3+^ spin state from low-spin (LS: *t*_2g_^6^*e*_g_^0^) to a higher spin (IS: *t*_2g_^5^*e*_g_^1^). The strong hybridization between Co 3*d* states of *e*_g_ symmetry and oxygen 2*p* states promoted the formation of broad σ* bands, which crossed over the *E*_F_ and gave rise to the half-metal character [81]. Du and coworkers reached the same conclusion in Sr and Fe codop-LaCoO_3_ OER catalyst [82]. In this regard, switching from 3 to 4*d* and 5*d* metals, the orbitals will become spatially more extended, increasing the covalency of the M–O bond but also decreasing the on-site Coulomb interaction [89].

#### Other Descriptors

**Fig. 8:**
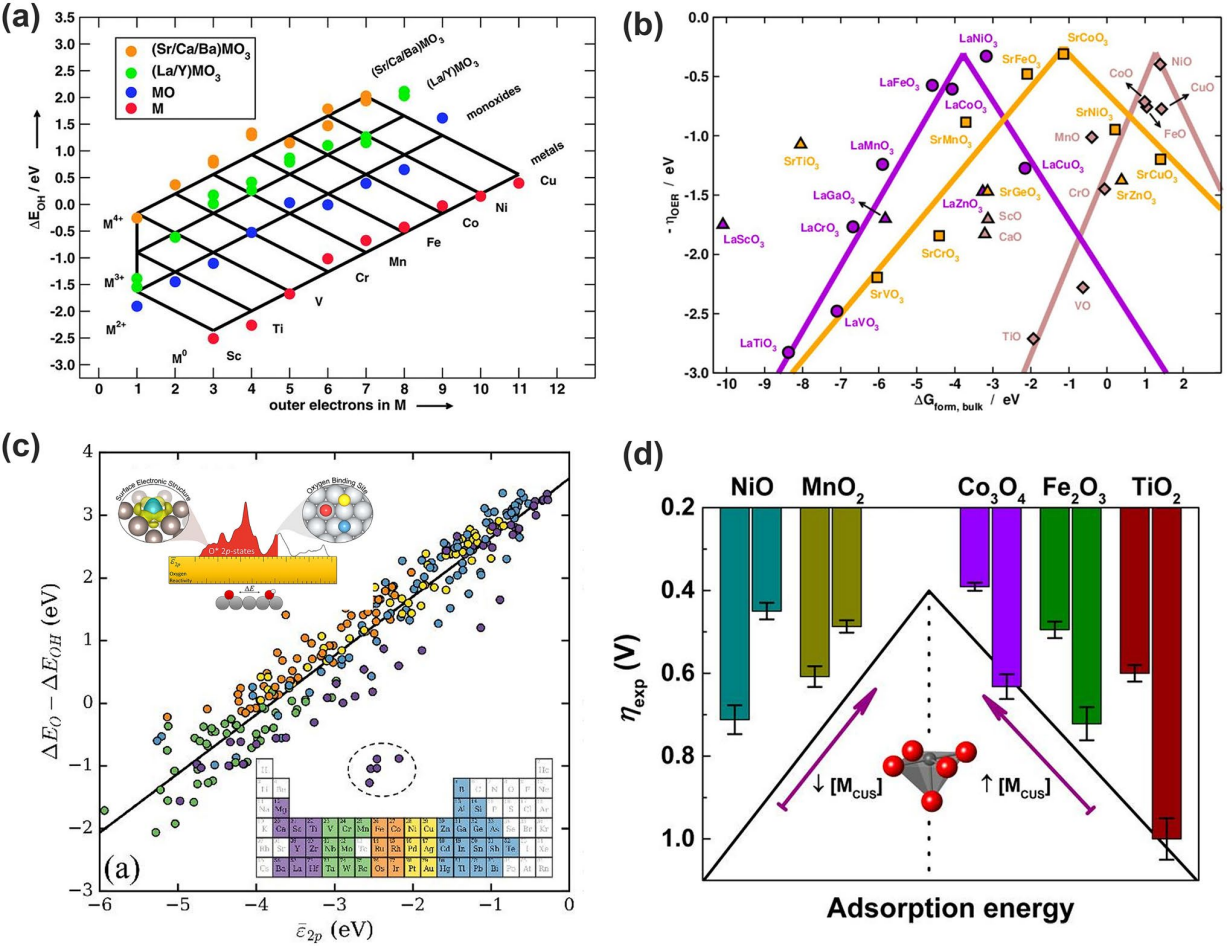
Descriptors correlated with the oxygen electrolysis catalytic activity. **a** Adsorption-energy grid of *OH on metals (red), monoxides (blue), La/Y perovskites (green), and Sr/Ca/Ba perovskites (orange), as a function of the number of outer electrons [90]. Copyright © 2013 Royal Society of Chemistry. **b** Volcano-type activity plot for LaMO_3_ (violet circles), SrMO_3_ (orange squares), and MO (brown rhomboids) [93]. Copyright © 2015 American Chemical Society. **c** Correlation between 2*p* and reactivity of surface oxygen atoms adsorbed at various metal and metal-oxide surfaces [94]. Copyright © 2019 Elsevier **d** Overview of the overpotential dependence on surface *M*_CUS_ for TMOs. The *η*_exp_ for Fe_2_O_3_ refers to current density of 1 mA cm^−2^, while that for the remaining TMOs corresponds to current density of 10 mA cm^−2^ [95]. Copyright © 2016 American Chemical Society. (Color figure online)

*(1) Outer electron numbers*. Otagawa and coworkers first employed the number of transition metal *d* electrons as a descriptor in their analysis of ABO_3_ electrocatalysts. They found the OER overpotential trended inversely with the enthalpy of hydroxide formation and *d*-electron number. Thus, it was concluded that the number of *d* electrons was the primary influence on the OH* bond strength via the occupancy of the metal-OH antibonding levels [66, 67]. Calle-Vallejo and coworkers later theoretically demonstrated that the binding strengths of possible OER intermediates scaled with the number of *d* electrons and oxidation state [51, 90–92]. As shown in Fig. 8a, the trends in adsorption energies of the intermediates of the OER on transition metals and their oxides are smoothly captured in terms of the number of outer electrons. Here, the outer electrons referred to the number of valence electrons remaining on the metal atom upon oxidation. This unique descriptor permits the construction of predictive adsorption-energy grids and explains the existence of scaling relationships among transition metals and their oxides [90].

*(2) Bulk Thermochemistry.* Bulk thermochemistry has long been used to describe the trends in catalytic activity of oxide surface [91, 93]. Following the *Outer electron numbers* descriptor, Calle-Vallejo and coworkers later found that bulk thermochemistry scaled similarly as surface adsorption energetics with the number of outer electrons of the TMs in oxides [93]. This correspondence applied to a wide number of TMs and was responsible for the linear relationship between bulk and surface properties that enables the construction of volcano-type activity plots, and rationalizes the trends in catalytic activity through variations in bulk thermochemistry (Fig. 8b). The volcano curves demonstrate that the majority of the oxides lie on their left legs; thus, the most active compounds tend to be the least stable ones. Such volcano plots provide direct estimations of the chemical stability of the oxides, which is paramount in the search for catalysts with good trade-offs between activity and stability under reaction conditions [93].

*(3) Average O-2p-State Energy (*${\overline{\varepsilon }}_{2p}$*).* Unlike most descriptors that consider the surface of the catalyst, Dickens and coworkers examined the reactivity and electronic structure of adsorbed oxygen [37, 94]. Figure 8c shows the correlation between the average O 2*p*-state energy, ${\overline{\varepsilon }}_{2p}$, and Δ*E*_O_ − Δ*E*_OH_ (namely, the energy required to deprotonate adsorbed OH to create adsorbed O) for oxygen atoms at various metal and metal oxides. It is apparent that bound oxygen atoms with higher-lying 2*p*-states have higher Δ*E*_O_ − Δ*E*_OH_, that is, stronger affinities towards binding hydrogen. In general, oxygen atoms with higher coordination to metal atoms tend to have weaker affinities toward binding hydrogen. The near-unity slope in Fig. 8c indicates that variations in ${\overline{\varepsilon }}_{2p}$ translate directly into variations in Δ*E*_O_ − Δ*E*_OH_ [94].

*(4) Coordinatively Unsaturated Metal Cation.* Tao and coworkers identified coordinatively unsaturated metal cations (M_CUS_) as a surface reactivity descriptor for the OER of TMOs [95]. In general, surface reactivity of a given TMO increases monotonically with the density of M_CUS_, and thus, the increase in M_CUS_ improves the catalytic activity for weak-binding TMOs (right line in Fig. 8d) but impairs that for strong-binding ones (left line in Fig. 8d). The electronic origin for this descriptor can be explained as that the energy of the highest occupied *d*-states relative to the Fermi level (*E*_d_–*E*_F_) serves as an electronic structure descriptor for the surface reactivity. This model is partially analogous to the *d*-band theory where the adsorption energy variations on TMs are mainly raised from the interaction of adsorbates with *d*-states of metal atoms; the difference is that the energy of highest occupied *d*-states relative to the Fermi level (*E*_d_–*E*_F_) determines the bond strength of adsorbates on TMOs.

Overall, the OER activity is principally estimated through the adsorption energy of intermediates on the catalyst surface. This universal, but theoretically deduced, descriptor was then reified into variable electronic or geometric parameters of electrocatalysts that can be obtained experimentally to establish the correlation with the OER performance trend. However, it is worth noting that each sub-descriptor mentioned above normally has its own special application domain. *d*-band theory describes many reactions when using transition metals and their alloys as catalysts; nonetheless, it fails to embrace catalyst surface information and is not effective to non-metallic catalysts. [43, 46] Though *e*_g_ occupancy model achieves great success by virtue of its tuning flexibility in describing the OER activity of various electrocatalysts, its ionic nature is unable to effectively capture the sharing of electrons along the metal–oxygen bond [78]. Metal–oxygen covalency model appears to be more powerful because it broadly not only involves the M 3*d* band and O 2*p* band, but also tackles with the energy difference between them (*ε*_3d-2p_). All of them are decisive parameters that give the electronic structure of a catalyst. It needs to take cautions in selecting an appropriate one for TMO catalysts under metal–oxygen covalency model. For example, O 2*p* band works for semimetal oxides, but is not effective for semiconducting oxides [79]. When considering charge transfer/electronic conductivity in designing OER catalyst, ε_3d-2p_, will be the first choice to correlate with OER activity. To the end, it is still challenging to deal with different catalysts using one experimental descriptor. The cooperative actions among descriptors should be taken into account to more precisely identify the catalytic activity and design advanced catalysts.

## OER Beyond Scaling Relations

All aforementioned descriptors are useful in screening the catalytic activity and designing more efficient catalysts; however, they remain effective under the usual scaling relationship that gives rise to a large intrinsic overpotential. What remains as a relevant question is how much further adsorption energies can be tuned to correspond to the very top of the volcano plot [1, 96]. Another insight suggested by these descriptors for further improving OER catalysts beyond the state-of-the-art is to break the robust scaling relationship between the binding energies of OH* and OOH* [97]. For example, one basic idea is to engineer active sites that selectively stabilize the latter. Essentially, this boils down to strengthening surface oxygen’s affinity toward hydroxyl without affecting its affinity toward atomic hydrogen [42, 97]. Recent advances majorly include: (1) constructing multiple functional centers that will be selectivity active for different intermediates to independently optimize the binding energies; (2) applying external physical effects like strain, nanoscopic confinement and magnetic field to separately tune certain intermediate; and (3) lattice oxygen participated OER mechanism.

### Constructing Multiple Functional Centers

The conventional concerted OER process was established on the redox reactions of a sole active cation. The interdependence between adsorption energies of intermediates reduces the degrees of freedom available for catalyst optimization [98]. Constructing multiple active centers would be a viable pathway that allows for the separate optimization of different intermediates, such as the recombination of oxygen adsorbates to form O_2_ or the dissociation of water [51]. Recent experimental and theoretical advances evidenced that the second active center could be either the TM cation or the non-metallic anions like C or the lattice oxygen.

#### Modified Active Centers Enabling O–O Direct Coupling

**Fig. 9:**
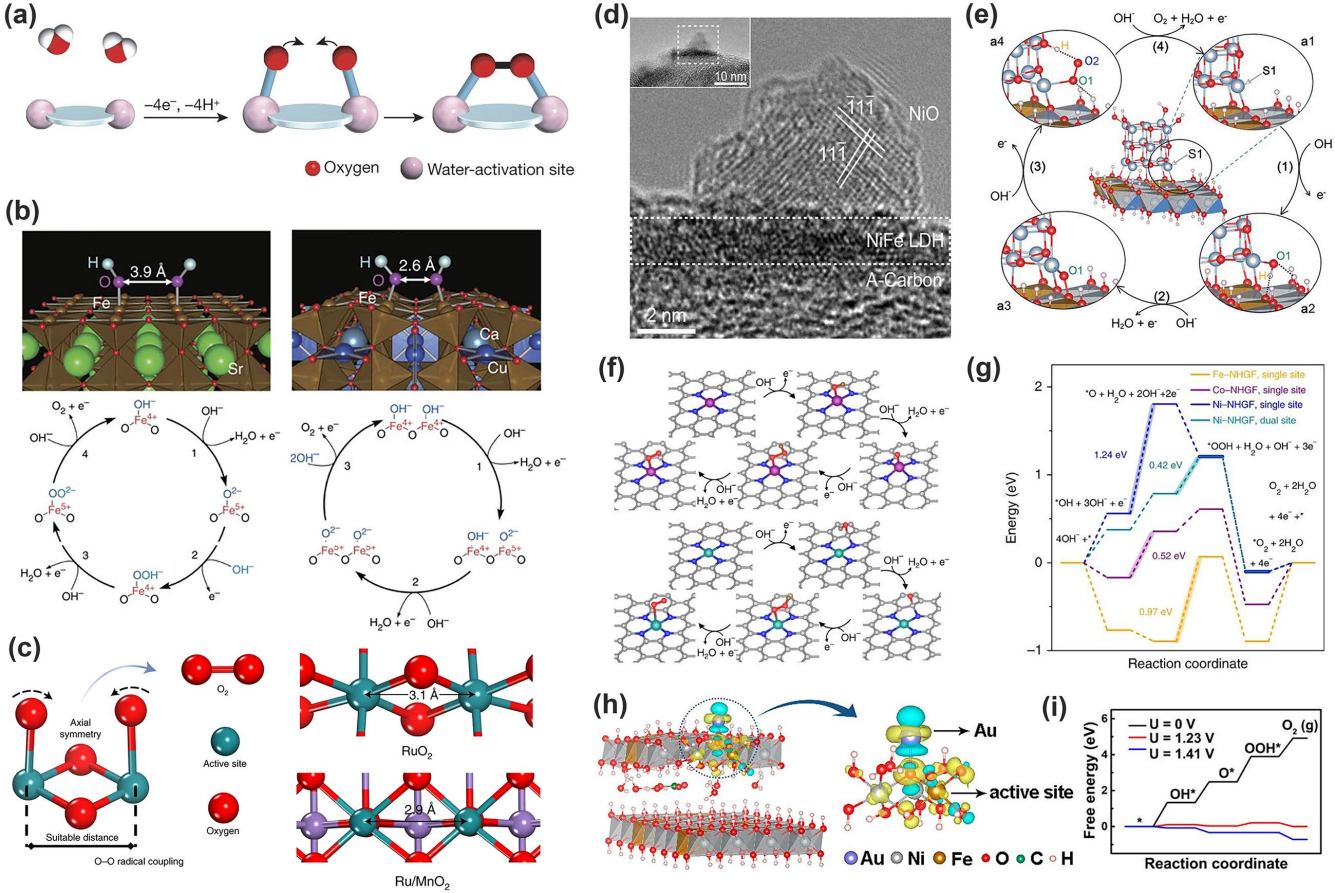
Oxygen generation through multiple functional centers. **a** Adjacent water-activation sites to promote intramolecular O–O bond formation [101]. Copyright © 2016 Springer Nature. **b** OH^−^ adsorbates on Fe-terminated (100) planes of SFO (left) and CCFO (right) for Fe-mediated route [83]. Copyright © 2015 Springer Nature. **c** O–O radical coupling promoted by symmetric dual active sites [105]. Copyright © 2021 Springer Nature. **d** HRTEM images of cross-section of LDH nanosheets combined with NiO NPs. **e** Schematic illustration of the OER pathway at S1 site of the NiO/NiFe LDH intersection [109]. Copyright © 2019 Wiley. **f** Proposed reaction scheme with the intermediates having optimized geometry of the single-site and dual-site mechanisms towards OER. **g** Free energy diagram at 1.23 V for OER over Fe-NHGF, Co-NHGF and Ni-NHGF with a single-site mechanism, and Ni-NHGF with a dual-site mechanism [111]. Copyright © 2018 Springer Nature. **h** Differential charge densities of NiFe LDH with and without Au atom when one O atom is adsorbed on the Fe site. **i** Free energy diagram for the OER at different potentials on the surface of ^s^Au/NiFe LDH model [112]. Copyright © 2018 American Chemical Society

The O–O bond formation is considered as the rate-determining step (RDS) in the water oxidation reaction. As commonly observed in the natural photosystem II where the Mn_4_Ca clusters rationalize the four-electron oxidation of water, a multinuclear core typically is advantageous when reactions involve multi-electron transfer [99–103]. In these molecular cluster catalysts, the two neighboring metal centers with a suitable distance undergo *OH deprotonation to produce two metal-oxo species, which then will couple to release a O_2_ molecule (Fig. 9a) [41, 101]. Analogously, this concept has been successfully adopted in heterogeneous electrocatalysis for the OER. Yagi and coworkers proposed the direct O–O formation in perovskite CaCu_3_Fe_4_O_12_ (Fig. 9b). In the regular SrFeO_3_ perovskite (left in 9b), the OER proceeds with the conventional AEM. OH^−^ is bound to B-site Fe ions, in which the RDS is the formation of the O–O bond (reaction 2) or the subsequent deprotonation (reaction 3) [83, 101]. Notably, after the incorporation of Fe^4+^ and Cu^2+^ in CaCu_3_Fe_4_O_12_, Fe–O-Fe bonds were heavily bent to give a shortened oxygen–oxygen (connected to the nearest neighboring Fe ions) distance of ~ 2.6 Å, which then enabled the occurrence of the direct formation of the O–O bond (right in Fig. 9b). In contrast, the oxygen–oxygen distance in sample SrFeO_3_ is ~ 3.9 Å, too large to interact with each other and form oxygen molecules. In this proposed pathway, the deprotonation of the oxyhydroxide group to form peroxide ions was skipped, resulting in the acceleration of the reaction and a lower overpotential due to the absence of a scaling relation. The catalyst CaCu_3_Fe_4_O_12_ demonstrated exceptional OER performance comparable to or exceeding those of state-of-the-art catalysts such as Ba_0.5_Sr_0.5_Co_0.8_Fe_0.2_O_3−*δ*_ and the gold standard RuO_2_ [83]. Short oxygen–oxygen distance also exists in α-Mn_2_O_3_, where the direct O–O bond formation mechanism was thought to dominate [104]. Very recently, Lin and coworkers reported an electrocatalysts with Ru-atom-array patches supported on *α*-MnO_2_ for the OER, where Ru atoms substituting surface Mn atoms formed small and periodically arranged ensembles. The interatomic Ru–Ru distance in Ru/MnO_2_ (2.9 Å) is shorter than that in RuO_2_ (3.1 Å), facilitating direct O–O radical coupling for O_2_ evolution (Fig. 9c). The proposed electrocatalyst 12Ru/MnO_2_ shows high activity (161 mV at 10 mA cm^−2^) and outstanding stability [105]. This pathway has been denoted as the oxide path mechanism (OPM) for heterogeneous catalysis [99, 101, 106–108]. It is worth noting that this mechanism involves only O* and HO* species as intermediates without the generation of *OOH. Therefore, OER electrocatalysts proceeding with OPM pathway can break the scaling relationship without sacrificing stability. The stringent challenge to enable the OPM is the geometric configuration of metal active sites.

#### Constructing Heterostructures and Heteroatoms

Separating *OH and *OOH on two different active sites would be expected to break the scaling relationship. An apparent way is to construct a heterostructure with fine interface engineering to ensure that the adsorption strength of each intermediate can be tuned independently. Gao and coworkers demonstrated the feasibility of this strategy in a NiO/NiFe layered double hydroxide heterostructure (Fig. 9d). In brief as shown in the proposed catalytic cycle (Fig. 9e), after OH^−^ groups get adsorbed on S1, the O and H atoms in *OH form two hydrogen bonds with hydroxyl and lattice O in LDH, respectively. Then, *OH deprotonates to give rise to *O on both S1 and LDH. Subsequently, *OOH forms with *H captured by the lattice O in LDH and *OO adsorbed on S1, respectively. Meanwhile, one hydrogen bond is formed between *OO and the hydroxyl in LDH. Finally, when OH^−^ captures *H and produces H_2_O, O_2_ is released. Based on the above, heterostructures exhibits two unique characteristics: (a) One or two additional chemical bonds always assist Ni cations (S1) in adsorbing intermediates; (b) The additional chemical bonds are different for each intermediate and vary dynamically during the whole OER process. As such, the binding energy of each intermediate can be adjusted independently, which provides an opportunity to break the scaling relationship [109].

Another approach is to incorporate heteroatoms in the crystal structure or on the surface by intervening in transition metal mediated catalysis with introduction of a proton acceptor to additionally stabilize *OOH. Halck and coworkers theoretically demonstrated that incorporation of Ni or Co into the surface on ruthenia could activate a proton donor–acceptor functionality on the conventionally inactive bridge sites (for *OOH) [110]. The calculation predicted that the optimized catalysts could achieve an overpotential of 0.1 and 0.25 V, much smaller than that for the conventional catalysts (0.37 V). However, the experimental results were much less significant than the theoretical predictions. It was believed that the clustering tendency violated the theoretical assumption. The recent advances in single atom catalysts (SACs) made the heteroatom structure possible to adjust the adjacent environments of the active centers. Fei and coworkers reported the synthesis of single-atom MN_4_C_4_ moieties (M = Fe, Co, Ni) for the OER, where both M and C atoms are possible adsorption sites for the different intermediates (Fig. 9f) [111]. Whether the C atoms participate in the OER process or not depends strongly on the number of *d* electrons of the metal in MN_4_C_4_ moieties. Specifically, for Fe and Co, the adsorption strength of all intermediates on the M site is stronger than that on the C site, and therefore, the adsorption-energy scaling between intermediates cannot be circumvented. On the other hand, for Ni, both intermediates of *O and *OH prefer to adsorb on the C site, whereas *OOH is favorably adsorbed on the M site, leading to circumvention of the scaling relationship (Fig. 9g). As a result, the NiN_4_C_4_ samples exhibited the best intrinsic activity, followed by FeN_4_C_4_ and CoN_4_C_4_ samples. Zhang and coworkers constructed single atom Au on NiFe layered double hydroxide (LDH) which revealed a sixfold enhancement for OER compared to pure LDH [112]. Upon the formation of Au = O bond (Fig. 9h), the spatially redistribution of *d* orbitals gives rise to the charge density around the ring region of Au parallel to the surface direction. The integrated charge density difference yields a net Au-to-LDH charge redistribution of 0.32 e, which transfers to surrounding O, Ni and Fe atoms, thus facilitates the adsorption of OH^−^ and modifies the adsorption energies of O* and OOH* intermediates, resulting in low overpotential in the rate-limiting step from O* to OOH*. The free energy diagram for OER (Fig. 9i) shows the RDS is the formation of OOH* from O* with a small overpotential of 0.18 V. Of significance, the corresponding binding energies Δ*E*_OH*_ and Δ*E*_OOH*_ for Au decorated LDH sample are 0.93 and 3.46 eV, resulting in favorable Gibbs energies for four elementary steps, i.e., $\Delta G_{1}^{0} = 1.33 \;{\text{eV}}$, $\Delta G_{2}^{0} = 1.15 \;{\text{eV}}$, $\Delta G_{3}^{0} = 1.41 \;{\text{eV}}$ and $\Delta G_{4}^{0} = 1.03 \;{\text{eV}}$ at standard conditions, which is very close to the thermochemically ideal OER process (Δ*E*_OH*_ = 0.86 eV and Δ*E*_OOH*_ = 3.3 eV, with $\Delta G_{1}^{0} = \Delta G_{2}^{0} = \Delta G_{3}^{0} = \Delta G_{4}^{0} = 1.23 \;{\text{eV}}$) [37, 112].

### Extrinsic Actions

#### Lattice Strain

**Fig. 10:**
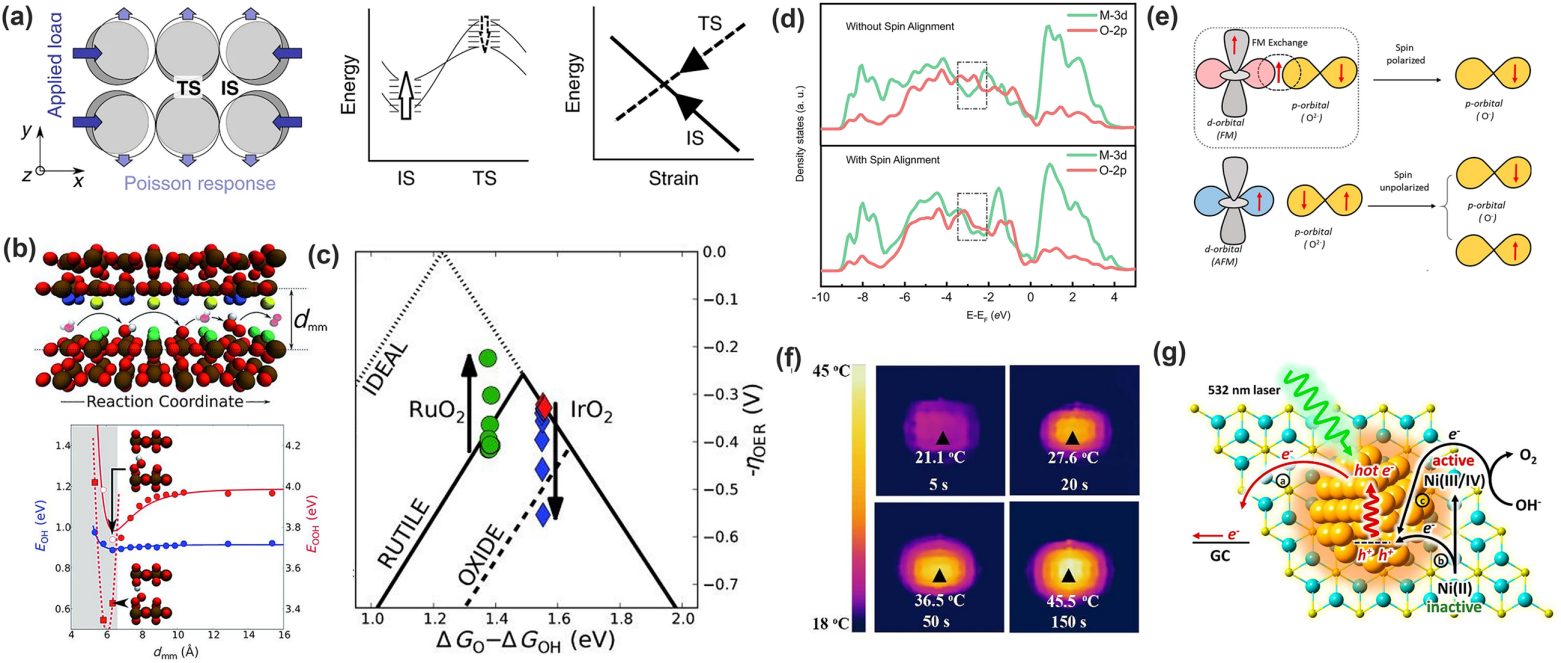
Extinct actions on the OER to potentially break the scaling relations. **a** Applying uniaxial compression to the surface. When qualitative picture of the energy level changes of the IS and TS with the applied loading, a violation of transition-state scaling is expected [117]. Copyright © 2018 Springer Nature. **b** Adsorption energies of OER intermediates (HO*, and HOO*) as a function of channel width for the OER relevant surface. **c** Volcano plot of the overpotential for OER; Arrows indicate trends in data as d_mm_ decreases [118]. Copyright © 2015 Wiley. **d** The projected density of states (PDOS) of CoFe_2_O_4_ without and with spin alignment. **e** Schematic of spin-exchange mechanism for OER [122]. Copyright © 2021 Springer Nature. **f** Infrared images of NFO/NF under NIR irradiation. [125] Copyright © 2021 National Academy of Sciences. **g** Schematic electron transfer paths likely to occur in the Ni(OH)_2_-Au electrode under 532 nm laser irradiation responsible for the OER catalysis [129]. Copyright © 2016 American Chemical Society

Introducing lattice strain is one of the possible approaches with external forces to circumvent the scaling relations. Lattice strain, either comprehensive or tensile, can alter the surface electronic structure by modifying the distance between surface atoms [113]. Based on *d*-band theory, tensile strain leads to stronger oxygen binding energy; conversely, comprehensive strain leads to weaker binding [113–115]. Wang and coworkers reported to tune the catalytic activity of Pt catalyst by controlling its lattice strain [113]. Their calculation showed that change ranges of Δ*G*_OH_ and Δ*G*_OOH_ are the same, whereas Δ*G*_O_ has a stronger dependence caused by its threefold coordination to the surface. Very similarly, Xie and coworkers, with N-doped graphene, theoretically demonstrated that tensile strain tends to stretch and break N–C* bond, which enhances the adsorption of *O while leaving that of *OH and *OOH unchanged [116]. Based on the results above, it appears that the O* adsorption can be controlled individually to potentially break the scaling relations among *O, *OH, and *OOH. Recently, Khorshidi and coworkers systematically described how strain can break this constraint by employing a mechanics-based eigenstress model to rationalize the effect of strain on adsorbate-catalyst bonding. In brief, when applying a uniaxial compression to a catalyst surface (Fig. 10a), if the energy level changes of the initial state (IS) and transition state (TS) resonate with the applied strain, the IS and TS are no longer correlated under applied strain, and the significantly decreased net reaction barrier upon strain can be expected to break scaling relation [117].

#### Nanoscopic Confinement

This concept was introduced by Doyle and coworkers who hypothesized that a 3D nanoscopic channel would be able to provide a confined reaction environment that enables selective interaction between intermediates and catalyst (Fig. 10b) [118]. With rutile oxides for the OER as examples, they analyzed the bond formation by varying the chemical environment and found that the pronounced stabilization occurs for *E*_OOH_ due to the hydrogen bond formation between HOO* and the opposite bridge site (Fig. 10b). Together with OER volcano based on the scaling-limited model (Fig. 10c), this model predicted that the overpotential for RuO_2_, on the left leg, could decrease to roughly 200 mV at *d*_mm_ ≈ 7 Å, that is beyond the scaling limits. However, catalysts on the right leg, for example IrO_2_, were unaffected by confinement because the potential was limited by the deprotonation of HO* to O*.

#### Magnetic Field

Applying an external magnetic field is a viable approach to regulate the chemical reactions and thus the catalytic activity because (1) the electrochemical catalysis proceeds with charge transfer, and (2) most TMO catalysts display magnetic susceptibility that can be apparently impacted by magnetic field [119]. More insightfully in regard of the OER, the formation of O–O bond by breaking water molecules requires the spin conservation to yield the paramagnetic triplet state of molecular oxygen. Thus, spin polarization of the active catalyst surface, enabled by external magnetic field, may favor parallel spin alignment of oxygen atoms during the reaction to improve the efficiency of the process [120, 121]. Most recently, Garcés-Pineda and coworkers reported enhanced OER activity of magnetic oxides with an externally applied magnetic field [120]. Experimental data indicated that the magnetic field was affecting the reaction pathway, and the magneto-enhancement appeared to be proportional to the magnetic nature of the catalysts. Therefore, the highly magnetic TMO oxides like Fe–Ni oxides are the preferred spin-dependent OER catalysts. Later, Ren and coworkers further concluded that magnetic field effect worked on the ferromagnetic ordered catalysts (CoFe_2_O_4_ spinel) as the spin polarizer for spin selection, but did not apply to non-ferromagnetic catalysts [122]. Meanwhile, the authors pointed out that the key step of spin-polarized OER occurred at the first electron transfer step, where coherent spin exchange between the ferromagnetic catalyst and the adsorbed oxygen species enabled a fast kinetics under the magnetic field. As a result, the first electron transfer step was no longer the RDS. Analogously, Zhang and coworker also found that the magnetic field-induced spin-polarized kinetics was more distinct in the case that the first electron transfer step is the RDS during the OER [123]. With the projected density of states (PDOS) analysis, Ren and coworkers suggested that the spin alignment enabled a stronger 3*d*-2*p* hybridization in catalyst (Fig. 10d) and gave a higher spin density on the oxygen atoms. Then, the concomitant increment of the 3*d*–2*p* hybridization associate with FM ligand hole facilitated FM exchange between the ferromagnetic catalyst and the adsorbed oxygen species with smaller electron–electron repulsion (Fig. 10e), which gave rise to the improved spin conductivity and decreased bonding energies, making the first electron transfer step no longer the RDS. It is noteworthy that Zhou and coworkers reported a thermal-differentiated superlattice created by localized magnetic heating in CoMn metal–organic-frameworks (MOFs) [124]. In this particular case, the magnetic heating effect was mainly localized at magnetic layers instead of heating-insulating organic linkers, and the lattice expansion of the whole system was limited effectivity. With the increase in MS operation time, the temperature of magnetic layer can reach ~ 480 K, but the remaining regions keep unchanged at room temperature. This heating localization resulted in the rearrangement of the spin electron occupation and demonstrated a spin-dependent reaction pathway toward unprecedented OER performance, where the catalytic activity of OER is authentically related with spin configuration of reactive site. Overall, it is still in its very early stage; nonetheless, it opens a new strategy to manipulate the spin polarization in magnetic oxide catalysts for promoting the OER and is encouraging more detailed studies to understand how the magnetic field-induced spin polarization affects the OER process.

#### Photo-Induced Oxygen Evolution

Recently, it was found that photo-assisted OER improvement could be realized in photothermal electrocatalysts through photothermal effect [34, 125]. As well known, increasing the temperature improves the catalytic activity. Photothermal electrocatalysts allow the heating generation under the illumination of visible or near infrared light. Lin and coworkers studied the photothermal effect improved OER activity in NiFe_2_O_4_ nanoparticles [125]. Under a NIR-light irradiation (808 nm), infrared imaging clearly indicated the temperature increase on NFO catalyst while leaving the electrolyte temperature unchanged (Fig. 10f). The photothermal effect was demonstrated not only to reduce the energy barrier and improve the OER kinetics, but also to lower the potential for forming the catalytically active species. On the other hand, photo-generated charge holes are also helpful to improve the OER activity. Different to the regular semiconductor photocatalysis where the semiconductors directly absorb light illumination and generate charge carriers for chemical reactions [126], photo-assisted OER mainly refers to that the OER electrocatalysts accept photo-holes from a light-active donor. The representative is plasmonic particles which generate hot charger carriers (electrons and holes) under the resonant light illumination [127, 128]. Ye and coworkers reported Au-nanoparticle-decorated Ni(OH)_2_ nanosheets for catalyzing the OER. Under the irradiation of a 532 nm laser, the plasmon-generated hot holes inject into Ni(OH)_2_ and improve the oxidation of Ni^2+^ to Ni^3+^/^4+^, thus leading to the boosted OER activity (Fig. 10g) [129]. It is noted that photo-generated holes on the OER pathways were barely investigated yet. From this aspect, more in-depth understanding is needed in the future.

### Lattice Oxygen-Mediated Mechanism (LOM)

#### Basics of the LOM

**Fig. 11:**
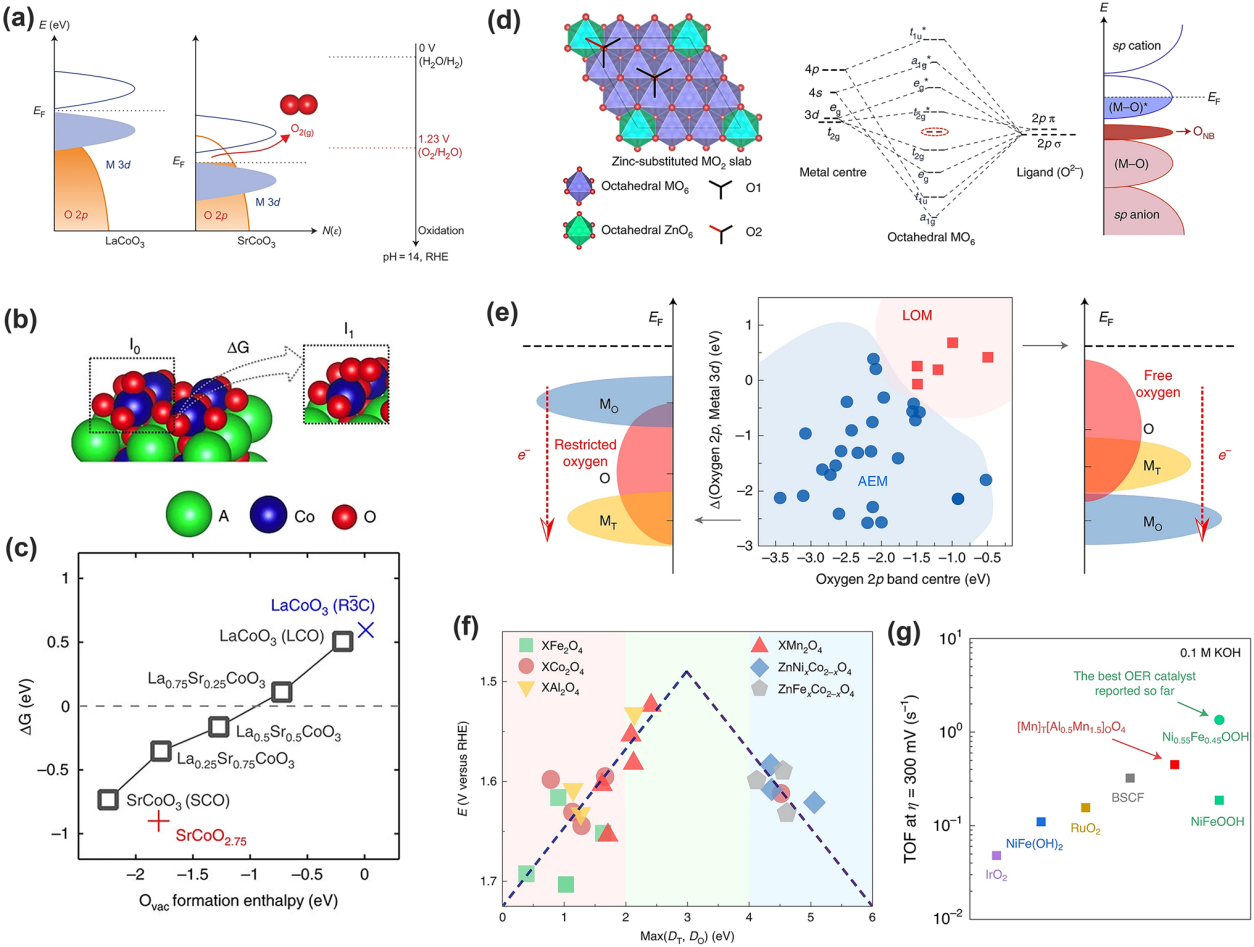
Lattice oxygen mediated mechanism (LOM) for OER. **a** Scheme of the band positions to trigger the LOM. The position of the O_2_/H_2_O redox couple at pH 14 is 1.23 V versus RHE [132]. Copyright © 2017 Springer Nature. **b** Surface configurations of the intermediate after AEM Step 1 (I_0_) and the one after LOM Step 1 (I_1_). **c** The free energy change of I_1_ over I_0_ versus the O vacancy formation enthalpy for the cubic La_1−*x*_Sr_*x*_CoO_3−*δ*_ [134]. Copyright © 2016 Springer Nature. **d** Model of zinc-substituted MO_2_ and Schematic formation of ONB by extrapolating the molecular orbital energy diagram for octahedral MO_6_ [139]. Copyright © 2019 Springer Nature. **e** An illustration of how the oxygen 2*p* band center, and the relative band centers between oxygen 2*p* and active metal 3*d*, co-regulate the reaction mechanism of OER on spinel oxides. **f** The experimentally observed reaction activity as a function of the calculated Max(*D*_T_, *D*_O_). **g** The intrinsic activity of the as-synthesized Al_0.5_Mn_2.5_O_4_ and other reference OER catalysts in 0.1 M KOH at an overpotential of 300 mV [131]. Copyright © 2020 Springer Nature

Recently, a new OER mechanism LOM has attracted intensive interests, which involves the participation of lattice oxygen for oxygen generation, and is able to rationalize the high intrinsic activity and surface reconstruction issues in some catalysts that were encountered and unexplained by conventional AEM. Compared to AEM (Fig. 2) that is based on cation redox chemistry, the LOM pathway cycle allows for the participation of lattice oxygen that must be active enough to escape and segregate from the lattice and interact with the adsorbed oxygen (*O) [130, 131]. In regular oxides, however, lattice oxygen is thermodynamically unfavorable given the deep location of the O 2*p* band caused by its large electronegativity (Fig. 11a) [130, 132, 133]. As shown as the rigid band diagram in Fig. 11a, the metal *d*-band of the metal oxides is generally located on the top of the oxygen *p*-band [134]. Anodic polarization causes an average increase in oxidation state of cations, then OER can be triggered when the O_2_/H_2_O redox potential aligns with the E_F_ of the oxide. This is the commonly accepted catalytic cycle under the AEM in which the metal cation evidently plays a dominant role by changing oxidation states and concomitantly the bond strengths with the intermediates accordingly [1]. To initiate the oxygen redox reactions (that is, the lattice oxygen becomes thermodynamically favorable for oxygen generation), the *E*_F_ has to move into the O 2*p* band and situates above the redox energy of the O_2_/H_2_O couple. Under this circumstance, *E*_F_ gets pinned at the top of the O 2*p* band, and electronic states near the E_F_ consist of substantial O 2*p* character. This electronic structure occurs in highly covalent oxides at which the overlap between M 3*d* and O 2*p* band is increased, i.e., the energy gap between O 2*p* center and *d* band center being minimized. Thus, it enables the electron transfer from lattice oxygen to the cation, resulting in the release of molecular oxygen and formation of oxygen vacancies [1, 132, 134–136]. In this regard, the later transition metal oxides with high metal–oxygen covalency are highly potential candidates for oxygen evolution with the LOM.

The release of lattice oxygen ligand from the catalyst surface is accompanied with the formation of oxygen vacancy (V_O_) that will be refilled and then act as a new active center. Mefford and coworkers presented a series of cobaltite perovskites and rationalized the roles of oxygen vacancies, M–O bond covalency, and redox activity of lattice oxygen through introducing Sr^2+^ substitution into La_1-*x*_Sr_*x*_CoO_3-*δ*_ [134]. With increasing Sr^2+^ substitution, higher Co–O covalency, more V_O_ and faster bulk oxygen ion diffusion rate were observed. When *x* > 0.5 (that is, ΔG starts to be negative, Fig. 11c), the formation of superoxide-like –OO(V_O_) adsorbates (*I*_1_) is remarkably energetically favorable compared to –O (*I*_0_) in conventional AEM, which enables the switch of OER from AEM to LOM (Fig. 11b, c) [134, 137]. Analogously, this group later introduced Sr^2+^ into Ruddlesden–Popper oxide La_0.5_Sr_1.5_Ni_1−*x*_Fe_*x*_O_4±*δ*_, and observed the participation of lattice oxygen as well [138]. It was believed that the Sr^2+^ introduction improved the oxidation state of Ni and thus enhanced Ni–O covalency and electronic conductivity. Direct experimental observation for lattice oxygen participating the OER process was achieved by Grimaud and coworkers with in situ ^18^O isotope labelling mass DEMS [132]. The authors further confirmed that metal–oxygen covalency not only regulated the OER activity but also determined the reaction mechanism. The OER could be switched to LOM when the electronic states near the E_F_ are filled with substantial O 2*p* character for highly covalent oxides.

Huang and coworker recently reported the possibility to trigger the LOM in oxyhydroxide through the inclusion of Zn^2+^ [139]. Different to perovskite, all three O 2*p* orbitals in the MOOH are engaged in M–O bond formation, while lacking the oxygen non-bonding (O_NB_) states that is essential for the formation of peroxo- or superoxo-like O–O dimers without the risk of structural destabilization [133, 140–142]. The authors proposed the incorporation of the low-valence and catalytically inactive Zn^2+^ into CoOOH to create accessible O_NB_ states and increase Co–O covalency simultaneously, the two necessary conditions to permit LOM in oxide-based electrocatalysts (Fig. 11d) [133, 140]. The theoretical and experimental results showed that creating oxygen holes in O_NB_ states along with the specific Zn–O2–Co–O2–Zn configuration is critical to regulating the OER mechanism; the OER proceeds via the LOM pathway only if the two neighboring oxidized oxygens can hybridize their oxygen holes without sacrificing metal–oxygen hybridization significantly.

Another representative case is spinel oxides AB_2_O_4_ with both A and B sites to be resided by transition metals, i.e., the co-existence of TMO_6_ and TMO_4_. Most recently, Sun and coworkers screened more than 300 spinel oxides and demonstrated two distinguishable energetical regions against the calculated oxygen 2*p* band center (ε_2*p*_) and the energy difference between the oxygen 2*p* and metal 3*d* band centers (Δ_O2*p*-M3*d*_): top-right suitable for LOM and bottom-left for AEM domains. ε_2*p*_ and Δ_O2*p*-M3*d*_ thus concurrently regulate the reaction mechanism of the OER (Fig. 11e) [131]. To achieve LOM, the oxygen 2*p* band center must be high enough (greater than − 1.75 eV) to guarantee its escape from the lattice, and meanwhile the oxygen *p* band level is higher than both cations for charge transfer from oxygen to cations. The authors further claimed that the OER activity on spinel oxides was intrinsically dominated by the covalency competition between tetrahedral and octahedral sites. The competition results in an asymmetric *M*_T_–O–*M*_O_ backbone where the bond with weaker metal–oxygen covalency determines the exposure of cation sites and the activity. Of significance, the correlation of the OER activity with the calculated Max(*D*_T_, *D*_O_) (namely, the covalency between tetrahedral/octahedral cations and oxygen) displays a volcano-like shape (Fig. 11f) [131]. Spinel oxides on the very left side have low OER activity since the strong covalency for both M–O bonds makes them difficult to generate active sites. While on the very right part, the bond breakage results in the low-covalent M–O bond, which have no unpaired electrons to adsorb the hydroxyl groups for OER. As a result, the bonds at the middle part of the volcano are optimized to be neither too strong nor too polarized. Following this line, Al_0.5_Mn_2.5_O_4_ with spinel distribution [Mn]_T_[Al_0.5_Mn_1.5_]_O_O_4_ was predicted to locate at the summit of the established volcano plot with remarkable activity for OER. The experimental results demonstrated an overpotential of 240 mV for [Mn]_T_[Al_0.5_Mn_1.5_]_O_O_4_ and an intrinsic activity comparable to the state-of-the-art catalysts (Fig. 11g). Of note, no surface reconstructions to amorphous or hydroxide structures were observed after long-time OER cycling [131].

#### Reaction Pathways in the LOM

**Fig. 12:**
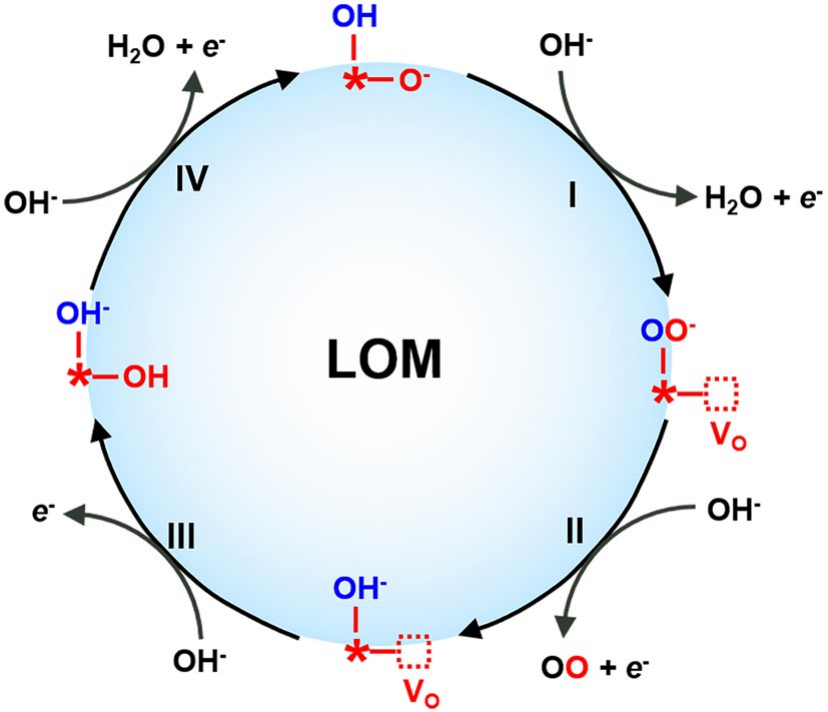
The OER catalytic cycle based on LOM. Here, * represents the surface-active TM cation, and the red dotted square represents the oxygen vacancy V_O_. The four elementary steps I, II, III, IV correspond to Eqs. 6–9

As discussed above, the LOM involves the lattice oxygen oxidization and the reversible formation of surface oxygen vacancies (Vo) in transition metal oxides. In a typical LOM catalytic cycle based on DFT calculations (Fig. 12), the dehydrogenation of *OH on the oxygen anion sites produces *OO species and V_O_ (Step I, Eq. 6), then the *OO species evolves back to *OH, while releasing O_2_ and electrons (Step II, Eq. 7). During this step, V_O_ is re-occupied by *OH and an adjacent surface lattice oxygen is protonated (Step III, Eq. 8). Finally, *OH is regenerated during the deprotonation process (Step IV, Eq. 9) [34, 134, 137, 138].

6 $$^{*}{\text{OH}} + {\text{OH}}^{ - } \to \left( {{\text{V}}_{{\text{O}}} + ^{*}{\text{OO}}} \right)^{\dag } + {\text{H}}_{2} {\text{O}} + {\text{e}}^{ - }$$

7 $$\left( {{\text{V}}_{{\text{O}}} + ^{*}{\text{OO}}} \right)^{\dag } + {\text{OH}}^{ - } \to {\text{O}}_{2} + \left( {{\text{V}}_{{\text{O}}} + ^{*}{\text{OH}}} \right)^{\dag } + {\text{e}}^{ - }$$

8 $$\left( {{\text{V}}_{{\text{O}}} + ^{*}{\text{OH}}} \right)^{\dag } + {\text{OH}}^{ - } \to \left( {{\text{OH}}^{ - } + ^{*}{\text{OH}}} \right)^{\dag } + {\text{e}}^{ - }$$

9 $$\left( {{\text{OH}}^{ - } + ^{*}{\text{OH}}} \right)^{\dag } + {\text{OH}}^{ - } \to ^{*}{\text{OH}} + {\text{H}}_{2} {\text{O}} + {\text{e}}^{ - }$$

The LOM mechanism eliminates the concerted proton-electron transfer steps as occurred in conventional AEM. It generates the oxygen through the direct coupling of O–O bond (Eq. 7) and does not involve the formation of *OOH, suggesting a potent strategy to break the scaling relationship in AEM. Two approaches could potentially trigger the LOM in TMO-based electrocatalysts: (1) activating the lattice oxygen by strengthening the metal–oxygen covalency and lowering the formation energy of the defect oxygen, and (2) intentionally including oxygen defects that function as the active centers for the LOM.

#### Experimental Observations Evidencing the LOM

As a newly proposed OER paradigm, convincing experimental observations are required to evidence the participation of lattice oxygen during the oxygen evolution. Below, the commonly employed techniques to confirm the LOM mechanism are highlighted.

1. in situ ^*18*^*O labelled electrochemical mass spectrometry (EMS).* The most straightforward methodology is to monitor the products with labelled catalysts or electrolyte during the OER. On-line electrochemical mass spectroscopy (OLEMS) is regularly employed to quantitatively determine the volatile products during the electrochemical reactions [130]. Grimaud and coworkers carried out OLEMS measurements of ^18^O labelled perovskites and first provided direct experimental evidence of the oxidation of lattice oxygen in highly covalent oxides (La_0.5_Sr_0.5_CoO_3−*δ*_ and LaCoO_3−*δ*_). Meanwhile, the results revealed that up to 37 monolayers of oxides (~ 14 nm) can be involved during the OER process [132]. Since then, ^18^O labelled EMS has been widely used as a powerful tool to monitor the participation of lattice oxygen [105, 130].
2. *Operando spectroscopy.* The operando spectroscopy techniques including infrared (IR) spectroscopy, Raman spectroscopy, X-ray absorption spectroscopy (XAS), and X-ray photoelectron spectroscopy (XPS) have been powerful tools for the investigation of electrocatalysts. The in situ use of these techniques for probing catalytic processes under reaction conditions gives accurate information regarding the dynamic changes occurring on electrode surface and deeper insight into basic mechanistic pathways [143]. As shown in the LMO catalytic pathways (Fig. 12), the lattice oxygen oxidation theoretically allows for the generation of ligand holes in O 2*p* orbitals, thus altering the electronic states. Also, the LMO process does not involve the formation of *OOH and generates the oxygen through the direct coupling of O–O bond. As such, *in-situ* monitoring of the catalyst surface during LOM-type OER with these operando techniques is viable to identify the reaction mechanism and the lattice oxygen redox chemistry in electrocatalysts [130]. Lin and coworkers carried out the operando synchrotron FT infrared (FTIR) spectroscopy because it is susceptible to the change of surface reaction intermediates. The formation of O–O bond at 1089 cm^−1^ could be clearly detected. Also, a unique peak at 1128 cm^−1^ is assigned to linearly bonded superoxol species, which are the intermediate before releasing O_2_ [105]. Several research groups have observed a shoulder peak of ~ 529 eV in O K-edge XAS spectra, that was assigned to positively charged oxygen species O^(2−*δ*)−^, and indicated the oxidation of lattice oxygen [144, 145]. Furthermore, coupling in situ Raman spectroscopy with ^18^O labeling, Hu and coworkers found that there existed a frequency variation at ~ 26 cm^−1^ with ^18^O isotope labeling, which was assigned to Co–O A_1g_ vibration and consolidated the lattice oxygen exchange with electrolyte [146, 147]. Since either AEM- or LOM- medicated OER process takes place on the catalyst surface, we believe those operando spectroscopy techniques that are sensitive to the variations of surficial geometrical and electronic structures will play a critical role in elucidating the underlying mechanism.
3. *Electrochemical pH-dependent OER activity*. Grimaud and coworkers found that the oxides capable of LOM reaction like La_0.5_Sr_0.5_CoO_3−*δ*_, Pr_0.5_Ba_0.5_CoO_3−*δ*_ and SrCoO_3−*δ*_ showed pH-dependent OER activity on the scale of the RHE, while the intrinsic OER activity of LaCoO_3_ through the AEM is independent of pH (Fig. 13a) [132]. This was ascribed to the presence of non-concerted proton-electron transfer step during OER, originating from the mismatch between electron transfer kinetics and hydrogen affinity at the oxide/electrolyte interface [79, 132, 148, 149]. In the case that the OER proceeds with the LOM, the substantial decrease in charge transfer energy accelerates electron transfer and weakens hydroxide affinity, decoupling them in PDS with pH-dependent activity. It is noted that the pH dependent OER activity was also observed in spinel ZnFe_x_Co_2-x_O_4_ oxides. However, that was believed to originate from the downshifted O *p*-band center relative to Fermi level induced by the spinel’s cation deficient nature and has nothing to do with the lattice oxygen oxidation [84, 150]. Therefore, the OER through the LOM can exhibit pH-dependent activity, nonetheless, the pH-dependent activity is not a decisive indicator for LOM reaction.

**Fig. 13:**
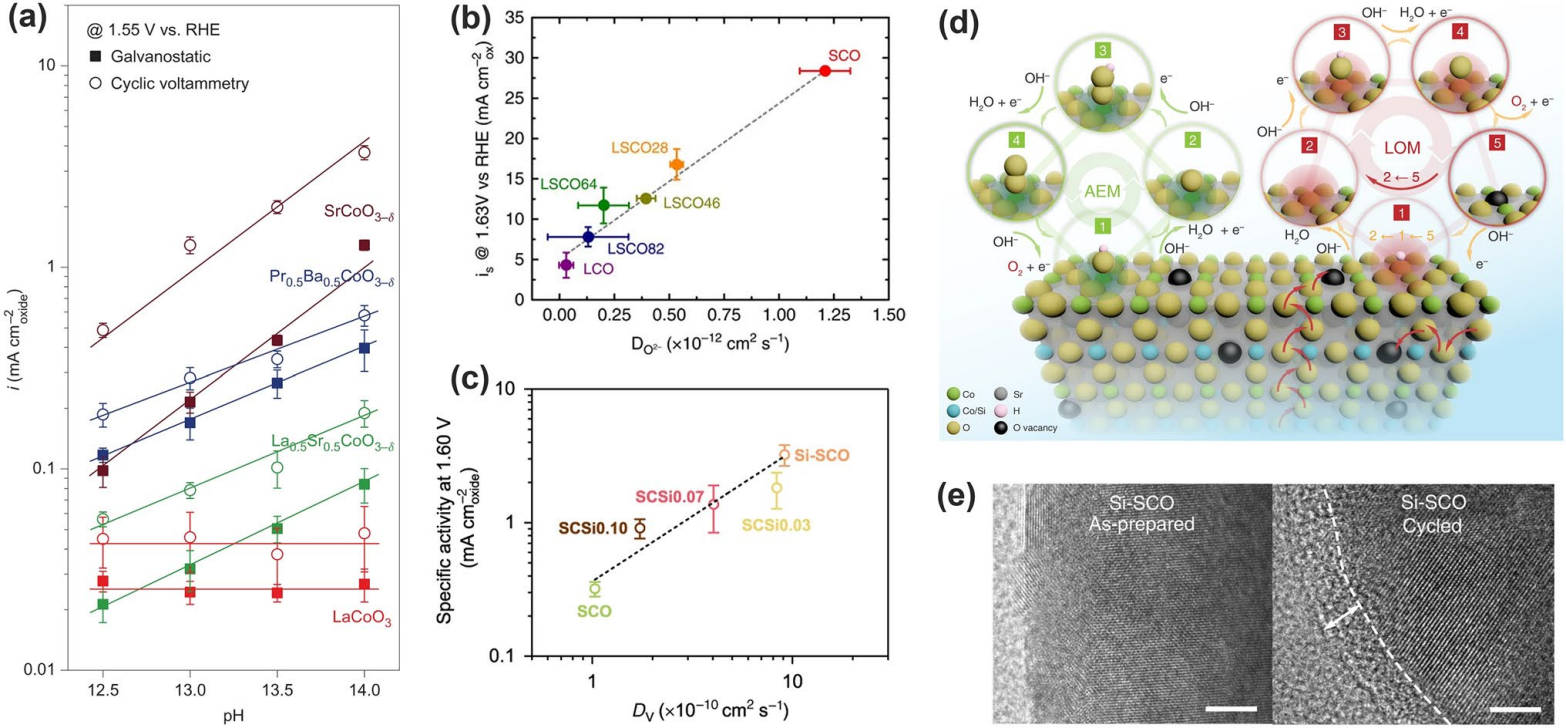
The pH-dependent activity, oxygen ion diffusion rate and surface stability for the OER in LOM pathways. **a** Specific OER activity of some perovskites at 1.55 V versus RHE after iR correction as a function of pH [132]. Copyright © 2017 Springer Nature. **b** Correlation of oxygen evolution activity with the oxygen ion diffusion rate in La_1−*x*_Sr_*x*_CoO_3−*δ*_ perovskites [134]. Copyright © 2016 Springer Nature. **c** Correlation of intrinsic OER activity in 0.1 M KOH with the oxygen anion diffusion rate. **d** A schematic illustration of the AEM and LOM reaction pathways on Si-incorporated strontium cobaltites. **e** HRTEM images for as-prepared and OER-Cycled Si-SCO [2]. Copyright © 2020 Springer Nature
4. *Surface Amorphization*. Given the participation of lattice oxygen for oxidation, migration and release under the LOM mediated OER, a high oxygen ions diffusion rate (*D*_O_) is vital to improve OER performance. Mefford and coworkers found that the catalytic activity towards the OER was strongly correlated with the oxygen diffusion rate in La_1−*x*_Sr_*x*_CoO_3−*δ*_ perovskite with a linear relationship (Fig. 13b) [134]. The SCO with the best OER activity through LOM pathway displays a *D*_O_ of ~ 1.2 × 10^–12^ cm^2^ s^−1^ is nearly two orders of magnitude higher than that of LaCoO_3_ (~ 3 × 10^–12^ cm^2^ s^−1^) with AEM for oxygen evolution. The *D*_O_ was further improved to 12.04 × 10^–11^ cm^2^ s^−1^ by introducing Si into SrCo_1-x_Si_x_O_3-*δ*_, which is ~ 12.8 times faster than SCO and correlated well with the tenfold improvement in intrinsic activity (Fig. 13c) [2]. Based on this correlation, Pan and coworker proposed that the surface oxygen vacancy left after oxygen evolution could be quickly replenished by oxygen ions diffusing from the bulk of the electrocatalyst, instead by the refilling of OH^−^/H_2_O from the electrolyte (Fig. 13d) [2]. In this regard, increasing the oxygen ion diffusion rate is able to facilitate the refilling of the oxygen vacancies left by surface lattice oxygen, consequently promoting the OER catalysis.

The involvement of lattice oxygen in the OER suggests that surface exchange kinetics could be a key catalyst design factor. Including bulk oxygen deficiency could switch the OER mechanism from AEM to LOM, which is beneficial for OER efficiency [137]. However, a large oxygen deficiency decreases bulk stability, especially in cases of high activity and could possibly result in cation leaching and surface amorphization, as in the case of BSCF and Si-SCO though with different refilling species (Fig. 13e). Under current LOM, therefore, balancing the surface oxygen vacancy refilling rate and the surface vacancy formation rate will be helpful. Given the goal to achieve higher activity, constructing surfaces which allow fast enough kinetics for oxygen vacancy refilling appears to be one direct means to address this issue [2, 89]. On the other hand, as demonstrated in some spinel oxides (Al_0.5_Mn_2.5_O_4_ in Fig. 11) and perovskites (CaCu_3_Fe_4_O_12_ in Fig. 9b), and oxyhydroxide (Zn incorporated MOOH in Fig. 11), the high OER activity could be achieved while keeping the stability without surface amorphization. Further understanding the underlying mechanism will be another direction for high-active OER catalyst design.

## Summary and Outlooks

As discussed above, the OER plays a vital role in determining the performance of ECS devices in utilization of renewable and sustainable energies. Therefore, developing high-efficiency and low-cost OER electrocatalysts to replace the noble metal-based Ru/Ir compounds is in urgent demand but remains a great technical challenge. In the last decade, we have witnessed lots of innovations in the catalyst fabrication and structure design, which was built on an in-depth understanding of the correlation between the catalyst and its catalytic behavior. Such fundamental understanding still needs to move forward before ideal catalyst could be reached. The OER activity is principally determined by the adsorption energy of intermediates on the catalyst surface. According to the conventional AEM pathway, the OER involves three oxygen-containing intermediates (*OH, *O, and *OOH). Tremendous research efforts have been made to build up the relationship between catalytic performance and catalysts’ electronic structures. For example, *d*-band theory, *e*_g_ occupancy, metal–oxygen covalency models have successfully manifested their effectiveness in interpreting the catalytic activity trend and guiding the design of more efficient catalysts. However, the intrinsic adsorption energy scaling relationship between intermediates imposes an overpotential of as large as 300–40 mV. Therefore, research emphasis should be placed on experimentally and theoretically explore innovative strategies to circumvent the energy scaling limit based on the previous understanding of AME-type OER. Significant advances have been achieved recently in (1) developing new OER mechanisms like the LOM and the OPM, (2) constructing multifunctional active centers to separate the adsorption of different intermediates, and (3) applying extrinsic physical actions that potentially impacts certain intermediates while not affecting others. Regardless of those significant accomplishments, challenges still remain in this area. To be specific, we bring up several perspectives for further advancing the development.

1. *Universal descriptor*. One powerful descriptor that bridges the structure and catalytic activity should be able to not only explain the current catalytic behavior but also precisely predict the catalytic trend, which is essential for the rational design of novel catalysts with superior activity. Unfortunately, each individual descriptor shows its limitation to accurately describing the structure–activity relationship. For example, *e*_g_ occupancy model was established on the ionic model and was unable to effectively capture the metal–oxygen covalency [78]. Moreover, the difficulty and sensitivity in determining the surface, sub-surface and bulk electronic is also an obstacle. Of very importance, to improve the descriptor-based design strategies, Hong and coworkers with statistical learning aggregated 101 observations in perovskites and identified five basic descriptor families, where covalency and electron occupancy hold the strongest influence on the OER activity [30]. The analysis suggested that combination of descriptors is necessary for concluding the best prediction. Besides, there remains a strong need to explore more physically meaningful approaches to fully understand the effects of chemistry on the OER activity. On the other hand, the most popular catalytic descriptors were developed in terms of bulk properties; whereas, catalytic reactions occur on the catalyst surface, the surface electronic structure information and the coordination chemistry should be fully taken into account in the future research. Overall, it is still a great challenge to discover a universal and measurable descriptor to accurately rationalize the structure–activity relationship [43].
2. *New OER paradigms*. The conventional OER paradigm (AEM) is hampered by the adsorption energy scaling relationship. The LOM skips the OOH* step and allows for the direct O–O formation with lattice oxygen and, thus, is a promising strategy to break the scaling relationship limit toward more efficient OER catalysts. Strengthened metal–oxygen covalency and oxygen vacancies are two prerequisites to permit the LOM in oxide-based electrocatalysts. Given the multimetallic positions in most oxides like perovskites, spinels, and LDHs, cation substitution has been a routine approach to modulate the OER toward the LOM pathway. In this regard, later TMs with large electronegativity will be potent dopants. Meanwhile, partially replacing oxygen with anions having larger electronegativity like F could also be able to improve the covalency and enable lattice oxygen oxidation during the OER [151, 152]. Additionally, introducing oxygen vacancy intentionally into the lattice matrix is highly expected to accelerate the lattice oxygen redox chemistry because the presence of Vo can tune the relative band position of TM and O 2*p* bands of the catalyst host and promote the metal–oxygen covalency [130]. Challenge remaining for the LOM is the long-term stability due to the cation leaching and surface amorphization. Constructing surfaces which allows fast enough kinetics for oxygen vacancy refilling appears to be one direction to address this issue [2, 89]. Of significance, the spinel Al_0.5_Mn_2.5_O_4_ [131], CaCu_3_Fe_4_O_12_ perovskites [83], and oxyhydroxide Zn incorporated MOOH [139] did not show surface amorphization while achieving high OER activity. Further understanding the underlying mechanism will be another direction for high-active OER catalyst design under the LOM to couple the stability and high activity. Analogous to the homogeneous oxo–oxo coupling mechanism, oxide path mechanism (OPM) has been proposed for heterogeneous catalysts [99, 101, 105]. Different to the LOM, the OPM pathway allows direct O–O radical coupling without the generation of oxygen vacancies and the participation of lattice oxygen. Therefore, the OPM is another highly promising candidate for further improving OER performance. However, the OPM has very stringent requirements for the geometric configuration of active sites. Engineering electrocatalysts to have symmetric dual-metal sites with appropriate atomic distances are technically challenging, but expected to promote O–O radical coupling with a low energy barrier [105].
3. *Operando characterizations*. Probing active sites with operando techniques will be a direct tool to identify the active center and more accurately understand the underlying reaction mechanisms. Given the surface reconstruction commonly observed during the OER because of the high applied potential on which the active sites are sensitive to the local environmental conditions [2, 58], this will be more useful to clarify what parameter governs the participation of lattice oxygen and what pathway is more thermodynamically favorable for oxygen refilling and oxygen ions diffusion and so on when combining in situ techniques, such as scanning probe microscopy, Raman spectroscopy, infrared spectroscopy, and X-ray absorption fine structure (XAFS) technique under electrochemical operation conditions [41]. For example, Grimaud and coworkers used OLEMS measurements and demonstrated the participation of lattice oxygen in perovskites [132]. Lin and coworkers employed operando synchrotron FT infrared (FTIR) spectroscopy and identified the distinctive absorption peak at 1128 cm^−1^ for linearly bonded superoxo species (metal-O–O) in 12Ru/MnO_2_ catalyst, which is the evidence for the direct O–O radical coupling pathway during the OER [105]. With operando X-ray absorption spectroscopy, Bai and coworkers revealed that the single-atom Co can be in situ transformed into Co-Fe dimeric moiety as the active center for the boosted OER activity [153]. As discussed above, the OER takes places on catalyst surface, which is very susceptible to the surrounding environments and cannot be tracked by ex situ technologies. Monitoring the catalyst surface with operando spectroscopic techniques during the reactions will be essential to shed light on the fundamental origin that triggers the OER and governs the reaction kinetics.
4. *Novel catalysts and advanced synthetic methodologies*. Despite the tremendous accomplishments in the last decades, there still exists a wide gap between the state-of-the-art OER catalysts and the ideal ones. Novel materials and structures along with the advanced.
  Overall, due to the sluggish reaction kinetics, the OER has long been the bottleneck for the electrochemical energy conversion and storage devices. This paper comprehensively reviewed the benchmark catalytic descriptors for OER electrocatalysis under the adsorption-energy scaling relationship among oxygen-containing intermediates. Given current achievements and limitations on the AEM, a summary of the physiochemical fundamentals and recent progress on the LOM, the OPM and other extrinsic actions to break the scaling relationship has been provided, targeting to help the researchers gain an in-depth understanding. Clarifying the new OER paradigms like the LOM and the OPM is in urgent demand to advance the further development by accelerating the OER kinetics and lowering the overpotentials. Integrating advanced operando techniques, novel synthetic methodologies and cutting-edge computational simulations will be essential to advance the OER fundamental research toward commercial ECS devices.
